# Adamantyl-Substituted Chalcone CA13 Induces Cytoprotective Autophagy and JNK-Dependent Apoptosis in Lung Cancer Cells

**DOI:** 10.3390/biom16010054

**Published:** 2025-12-30

**Authors:** Yuting Chen, Yaxin Liu, Jing Zhou, Tingting Bao, Jing Wang, Mingtao Ao

**Affiliations:** 1 School of Pharmacy, Hubei University of Science and Technology, Xianning 437100, China; 2 Institutes of Biomedical Science, Inner Mongolia University, Hohhot 010021, China; 3 Xiantao First People’s Hospital, The Affiliated Hospital of Hubei University of Science and Technology, Xiantao 433000, China

**Keywords:** chalcone, apoptosis, autophagy, JNK, lung cancer

## Abstract

Lung cancer remains a leading cause of cancer mortality worldwide, highlighting the need for novel therapeutics. Here, we designed and synthesized a series of adamantyl-substituted chalcones and identified CA13 as a lead compound with potent and selective antiproliferative activity against non–small cell lung cancer (NSCLC) cells. CA13 triggered both apoptosis and autophagy in H292 cells. Western blotting and confocal imaging confirmed the activation of complete autophagic flux, while inhibition of autophagy markedly enhanced CA13-induced apoptosis, suggesting a cytoprotective role of autophagy. Mechanistically, CA13 activated JNK phosphorylation in a dose- and time-dependent manner, and pharmacological blockade of JNK significantly attenuated apoptotic signaling. In vivo, CA13 effectively suppressed H292 xenograft tumor growth without apparent systemic toxicity. Collectively, these results demonstrate that CA13 exerts its antitumor effects through JNK-dependent apoptosis accompanied by cytoprotective autophagy, providing a promising structural framework for the development of chalcone-based anticancer agents targeting programmed cell death pathways.

## 1. Introduction

Lung cancer remains one of the leading causes of cancer-related mortality worldwide [1]. Despite significant advances in targeted therapies and immunotherapies, therapeutic resistance and disease relapse remain major challenges, underscoring the urgent need for novel small-molecule agents with potent antitumor activity and high favorable selectivity [2,3]. Non-small cell lung cancer (NSCLC) represents approximately 85% of all lung cancer cases, and most patients are diagnosed at advanced or metastatic stages, where curative surgical options are limited [4]. Therefore, the discovery of new therapeutic compounds capable of targeting key molecular pathways that drive cancer progression remains a critical objective in lung cancer research.

**Figure 1:**
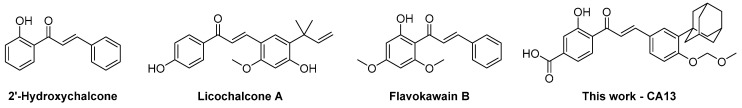
Chemical structures of representative chalcones.

Natural product-derived scaffolds continue to provide a valuable source of inspiration for anticancer drug discovery, among which chalcones (1,3-diphenyl-2- propen-1-one derivatives) have attracted particular interest due to their structural simplicity, ease of chemical modification, and broad range of biological activities [5,6,7,8]. Chalcones, characterized by their α,β-unsaturated carbonyl scaffold, are a class of naturally occurring flavonoids found in various edible plants [9]. They exhibit diverse pharmacological activities, including anti-inflammatory [10], antioxidant [11], and anticancer effects [12]. Increasing evidence suggests that the anticancer effects of chalcones are largely attributed to their ability to modulate various forms of programmed cell death, particularly apoptosis and autophagy [13,14,15,16]. Chalcone derivatives have been shown to provoke mitochondrial-mediated apoptosis through reactive oxygen species (ROS) accumulation, collapse of mitochondrial membrane potential, cytochrome c release, modulation of Bcl-2 family proteins, caspase activation and PARP cleavage [7,17,18,19]. Meanwhile, an increasing number of studies demonstrate that chalcones can also regulate autophagy: either by inducing autophagic flux or by inhibiting autophagosome degradation, thus influencing the delicate balance between cell survival and cell death [20,21,22,23,24,25,26]. For instance, 2′-Hydroxychalcone (2′-HC) (Figure 1) enhanced the autophagic levels and induced apoptosis in breast cancer cells [23]. Synthetic 2′-hydroxychalones induced apoptosis and autophagy in A549 lung cancer cells [24]. Similarly, Licochalcone A (Figure 1) and related chalcones triggered both apoptosis and autophagy while concurrently reducing cellular invasiveness, emphasizing the dual roles of these pathways in anticancer responses [25,26].

Autophagy, however, plays a dual role in cancer therapy. It may function as a cytoprotective mechanism, enabling tumor cells to survive metabolic stress and therapeutic insult, or conversely, act as a pro-death mechanism when excessively or aberrantly activated [27]. The biological outcome of autophagy modulation often depends on cancer type, treatment context, and dosage. Several studies have shown that chalcone derivatives can trigger autophagy as a survival mechanism, and that pharmacological or genetic inhibition of autophagy significantly enhances chalcone-induced apoptosis [26,28,29]. For example, blocking autophagy significantly increased flavokawain B-mediated cytotoxicity, indicating that flavokawain B induces protective autophagy in thyroid cancer cells [28].

In our continuing efforts to identify novel modulators of apoptosis and autophagy as potential anticancer candidates [30], we designed and synthesized a series of adamantyl-substituted chalcone derivatives. The introduction of the bulky hydrophobic adamantyl group was intended to enhance lipophilicity and facilitate stronger molecular interactions, while fine-tuning the electronic properties of aromatic substituents aimed to optimize biological activity. In this study, we characterized the anticancer effects of the most active compound, **CA13** (Figure 1), focusing on its ability to induce apoptosis and autophagy in lung cancer cells and evaluating its in vivo antitumor efficacy.

## 2. Materials and Methods

### 2.1. Chemistry

All the reagents were obtained from Bide Pharmatech Ltd. (Shanghai, China). The progress of the reaction was monitored by thin-layer chromatography (TLC) on pre-coated silica gel 60 F254 plates, and a UV light was used to visualize agent. ^1^H NMR (400 MHz) and ^13^C NMR (100 MHz) are obtained by Bruker Avance III 400 spectrometer (Bruker Corporation, Billerica, MA, USA). Tetramethylsilane (TMS) is used as the internal standard, and ppm is used as the unit to record the Chemical shift (δ). The terms s, d, t, q, m refer to singlet, doublet, triplet, quartlet, multiplet signals. The J value (Coupling constant) is expressed in Hertz (Hz). Mass spectra were obtained on a SHIMADZU LCMS-8040 spectrometer (Shimadzu, Kyoto, Japan) with ESI. Fourier transform infrared (FT-IR) spectra were recorded on a SHIMADZU IRAffinity-1 spectrometer. Melting points were determined using an SGW X-4 model apparatus (INESA, Shanghai, China).

#### 2.1.1. The Synthesis of 3-(Adamant-1-yl)-4-(Methoxymethoxy)-Benzaldehyde (**3**)

Compound **3** was prepared according to our previously reported procedure [30] with necessary modifications. To a solution of 4-hydroxybenzaldehyde **1** (1.22 g, 10 mmol) and adamant-1-ol (1.69 g, 11.1 mmol) in glacial acetic acid (10 mL) was added concentrated H_2_SO_4_ (0.65 mL, 12 mmol). The reaction mixture was stirred for 6 h at 70 °C. Upon completion of the reaction, the reaction mixture was quenched with ice water, and the precipitated solid was filtered, washed with water and petroleum ether, and dried to get **2**. Then, compound **2** (1.28 g, 5 mmol) was dissolved in acetone (30 mL). Chloromethyl methyl ether (MOMCl) (1.14 mL, 15 mmol) and anhydrous potassium carbonate (2.07 g, 15 mmol) were added and the reaction mixture was stirred at 60 °C for 12 h. After completion, the reaction mixture was filtered and the filtrate was concentrated under reduced pressure. The crude was recrystallized with ethanol to give **3**. White solid, yield 55%.

#### 2.1.2. General Procedure for the Preparation of Chalcones (**CA 5–13**)

A mixture of corresponding acetophenone (1 mmol) and benzaldehyde derivatives (1.1 mmol) in ethanol (10 mL) was added NaOH (10 eq.) and stirred at 70 °C for 12–24 h. Upon reaction completion, the reaction mixture was poured into ice water and neutralized with 1 N HCl. The solid was filtered and purified by column chromatography on silica gel to give the corresponding chalcones **CA 5–13**.

*(E)-3-hydroxy-4-(3-(4-hydroxyphenyl)acryloyl)benzoic acid (**CA-5**)*

A yellow solid, yield: 20.3%. mp 126−128 °C. ^1^H NMR (400 MHz, DMSO-d_6_) δ 8.55 (d, *J* = 2.3 Hz, 1H), 8.04 (dd, *J* = 2.1, 8.7 Hz, 1H), 7.75 (dd, *J* = 3.5, 9.3 Hz, 4H), 7.06 (d, *J* = 8.5 Hz, 1H), 6.85 (d, *J* = 8.8 Hz, 2H). ESI-MS: *m*/*z* calcd. for C_16_H_11_O_5_^−^ [M − H]^−^ 283.06, found 283.06.

*(E)-3-(3-adamantan-1-yl)-4-(methoxymethoxy)phenyl)-1-(4-fluoro-2-hydroxyphenyl)prop-2-en-1-one (**CA-6**)*

A yellow solid, yield: 45.8%. mp 148−150 °C. IR (KBr, cm^−1^): 2905, 2854 (aliphatic C–H, adamantyl); 1640 (C=O); 1572 (C=C, conjugated & Ar); 1492 (Ar C=C/C–H); 1234, 1134 (C–O–C); 991 (trans-CH=CH out-of-plane bending). ^1^H NMR (400 MHz, DMSO-d_6_) δ 12.83 (d, *J* = 7.5 Hz, 1H), 8.26 (t, *J* = 8.0 Hz, 1H), 7.81–7.85 (m, 2H), 7.75 (dd, *J* = 6.0, 7.5 Hz, 1H), 7.65 (d, *J* = 5.1 Hz, 1H), 7.11 (d, *J* = 5.7 Hz, 2H), 7.07 (dd, *J* = 6.0, 8.0 Hz, 1H), 5.34 (s, 2H), 3.47 (s, 3H), 2.13–2.08 (m, 9H), 1.80–1.75 (m, 6H). ^13^C NMR (100 MHz, DMSO-d_6_) δ 193.1, 162.8, 158.8, 146.5, 140.3, 138.8, 132.9, 129.3, 128.5, 127.8, 120.6, 119.8, 119.7, 117.8, 115.0, 94.3, 56.8, 40.5, 37.2, 37.0, 28.8. ESI-MS: *m*/*z* calcd. for C_27_H_30_FO_4_^+^ [M + H]^+^ 437.21, found 437.30.

*(E)-3-(3-adamantan-1-yl)-4-(methoxymethoxy)phenyl)-1-(4-chloro-2-hydroxyphenyl)prop-2-en-1-one (**CA-7**)*

A yellow solid, yield: 57.4%. mp 130−132 °C. IR (KBr, cm^−1^): 2910, 2852 (aliphatic C–H, adamantyl); 1640 (C=O); 1572 (C=C, conjugated & Ar); 1492 (Ar C=C/C–H); 1238, 1134 (C–O–C); 991 (trans-CH=CH out-of-plane bending). ^1^H NMR (400 MHz, CHLOROFORM-d) δ 13.17 (s, 1H), 7.94 (d, *J* = 15.3 Hz, 1H), 7.86 (d, *J* = 8.6 Hz, 1H), 7.56 (d, *J* = 2.0 Hz, 1H), 7.50 (dd, *J* = 2.0, 8.6 Hz, 1H), 7.45 (d, *J* = 15.3 Hz, 1H), 7.15 (d, *J* = 8.4 Hz, 1H), 7.05 (d, *J* = 2.0 Hz, 1H), 6.93 (dd, *J* = 2.0, 8.6 Hz, 1 H), 5.30 (s, 2 H), 3.55 (s, 3 H), 2.17–2.12 (m, 9 H), 1.82–1.78 (m, 6 H). ^13^C NMR (100 MHz, CHLOROFORM-d) δ 192.9, 164.3, 159.1, 146.8, 141.8, 139.3, 130.5, 128.1, 128.0, 127.7, 119.4, 118.7, 118.6, 117.1, 114.8, 94.1, 56.5, 40.5, 37.2, 37.0, 29.0. ESI-MS: *m*/*z* calcd. for C_27_H_30_ClO_4_^+^ [M + H]^+^ 453.18, found 453.30.

*(E)-3-(3-adamantan-1-yl)-4-(methoxymethoxy)phenyl)-1-(4-bromo-2-hydroxyphenyl)prop-2-en-1-one (**CA-8**)*

A yellow solid, yield: 50.8%. mp 135−138 °C. IR (KBr, cm^−1^): 2910, 2852 (aliphatic C–H, adamantyl); 1642 (C=O); 1572 (C=C, conjugated & Ar); 1492 (Ar C=C/C–H); 1236, 1131 (C–O–C); 991 (trans-CH=CH out-of-plane bending). ^1^H NMR (400 MHz, DMSO-d_6_) δ 12.78 (s, 1H), 8.21 (d, *J* = 8.4 Hz, 1H), 7.83–7.76 (m, 2H), 7.73 (dd, *J* = 1.7, 8.4 Hz, 1H), 7.62 (d, *J* = 1.5 Hz, 1H), 7.13 (d, *J* = 2.0 Hz, 1H), 7.10 (d, *J* = 8.4 Hz, 1H), 7.04 (dd, *J* = 1.8, 8.4 Hz, 1H), 5.33 (s, 2H), 3.46–3.44 (m, 3H), 2.11–2.07 (m, 9H), 1.78–1.73 (m, 6H). ^13^C NMR (100 MHz, DMSO-d_6_) δ 193.0, 162.5, 158.7, 146.3, 140.1, 138.8, 132.8, 129.2, 128.5, 127.8, 120.9, 120.0, 119.8, 117.8, 115.0, 94.3, 56.7, 40.4, 37.2, 37.0, 28.8. ESI-MS: *m*/*z* calcd. for C_27_H_28_BrO_4_^+^ [M − H]^−^ 495.12, found 495.25.

*(E)-3-(3-adamantan-1-yl)-4-(methoxymethoxy)phenyl)-1-(2-hydroxy-4-methylphenyl)prop-2-en-1-one (**CA-9**)*

A yellow solid, yield: 47.6%. mp 125−127 °C. IR (KBr, cm^−1^): 2910, 2846 (aliphatic C–H, adamantyl); 1648 (C=O); 1602 (C=C, conjugated & Ar); 1236, 1133 (C–O–C); 995 (trans-CH=CH out-of-plane bending). ^1^H NMR (400MHz, DMSO-d_6_) δ 12.98 (s, 1H), 8.23 (d, *J* = 8.3 Hz, 1H), 7.93–7.85 (m, 2H), 7.81–7.78 (m, 1H), 7.66 (d, *J* = 1.8 Hz, 1H), 7.14 (d, *J* = 8.6 Hz, 1H), 6.89–6.84 (m, 2H), 5.37 (s, 2H), 3.49 (s, 3H), 2.38 (s, 3H), 2.16–2.11 (m, 6H), 1.81–1.77 (m, 6 H). ^13^C NMR (100 MHz, DMSO-d_6_) δ 193.5, 163.0, 158.6, 148.0, 145.9, 138.7, 131.2, 129.0, 128.5, 127.9, 120.7, 119.1, 118.4, 118.2, 115.0, 94.2, 56.7, 40.4, 37.2, 37.0, 28.9, 21.9. ESI-MS: *m*/*z* calcd. for C_28_H_33_O_4_^+^ [M + H]^+^ 433.24, found 433.30.

*(E)-3-(3-adamantan-1-yl)-4-(methoxymethoxy)phenyl)-1-(2-hydroxy-4-methoxyphenyl)prop-2-en-1-one (**CA-10**)*

A red solid, yield: 44.3%. mp 135−137 °C. IR (KBr, cm^−1^): 2910, 2852 (aliphatic C–H, adamantyl); 1640 (C=O, chalcone); 1575 (C=C, conjugated & Ar); 1499 (Ar C=C/C–H); 1234, 1133 (C–O–C); 991 (trans-CH=CH out-of-plane bending). ^1^H NMR (400MHz, DMSO-d_6_) δ 13.58 (s, 1H), 8.31–8.26 (m, *J* = 9.2 Hz, 1H), 7.87–7.78 (m, 2H), 7.77–7.73 (m, 1H), 7.62 (s, 1H), 7.11–7.09 (m, *J* = 8.6 Hz, 1H), 6.56 (dd, *J* = 2.2, 9.0 Hz, 1H), 6.52 (d, *J* = 2.2 Hz, 1H), 5.33 (s, 2H), 3.85 (s, 3H), 3.45 (s, 3H), 2.12–2.07 (m, 9H), 1.78–1.74 (m, 6H). ^13^C NMR (100 MHz, DMSO-d_6_) δ 192.4, 166.3, 166.1, 158.5, 145.4, 138.7, 133.1, 129.0, 128.4, 128.0, 119.0, 115.0, 114.4, 107.8, 101.4, 94.3, 56.7, 56.2, 40.5, 37.2, 37.0, 28.8. ESI-MS: *m*/*z* calcd. for C_28_H_33_O_5_^+^ [M + H]^+^ 449.23, found 449.35.

*(E)-3-(3-adamantan-1-yl)-4-(methoxymethoxy)phenyl)-1-(2-hydroxy-4,6-dimethoxyphenyl)prop-2-en-1-one (**CA-11**)*

A red solid, yield: 40.6%. mp 168−170 °C. IR (KBr, cm^−1^): 2910, 2850 (aliphatic C–H, adamantyl); 1640 (C=O, chalcone); 1598 (C=C, conjugated & Ar); 1507 (Ar C=C/C–H); 1234, 1131 (C–O–C); 997 (trans-CH=CH out-of-plane bending). ^1^H NMR (400 MHz, CHLOROFORM-d) δ 7.89–7.76 (m, 2H), 7.53 (d, *J* = 2.2 Hz, 1H), 7.44 (dd, *J* = 2.1, 8.4 Hz, 1H), 7.12 (d, *J* = 8.4 Hz, 1H), 6.12 (d, *J* = 2.3 Hz, 1H), 5.98 (d, *J* = 2.4 Hz, 1H), 5.28 (s, 2H), 3.92 (s, 3H), 3.85 (s, 3H), 3.54 (s, 3H), 2.15–2.11 (m, 9H), 1.83–1.77 (m, 6H). ^13^C NMR (100 MHz, CHLOROFORM-d) δ 192.6, 168.4, 166.0, 162.5, 158.2, 143.2, 138.9, 128.8, 127.7, 127.3, 125.2, 114.7, 106.4, 94.1, 93.8, 91.3, 56.4, 55.8, 55.6, 40.6, 37.2, 37.1, 29.1. ESI-MS: *m*/*z* calcd. for C_29_H_35_O_6_^+^ [M + H]^+^ 479.24, found 479.35.

*(E)-3-(3-((3r,5r,7r)-adamantan-1-yl)-4-(methoxymethoxy)phenyl)-1-(2,4,6-trimethoxyphenyl)prop-2-en-1-one (**CA-12**)*

A yellow solid, yield: 47.6%. mp 185−187 °C. IR (KBr, cm^−1^): 2908, 2850 (aliphatic C–H, adamantyl); 1650 (C=O); 1598 (C=C, conjugated & Ar); 1507 (Ar C=C/C–H); 1235, 1133 (C–O–C); 997 (trans-CH=CH out-of-plane bending) ^1^H NMR (400 MHz, DMSO-d_6_) δ 7.50 (dd, *J* = 2.0, 8.6 Hz, 1H), 7.36 (d, *J* = 2.0 Hz, 1H), 7.15 (d, *J* = 16.0 Hz, 1H), 7.04 (d, *J* = 8.6 Hz, 1H), 6.79 (d, *J* = 16.0 Hz, 1H), 6.31 (s, 2H), 5.29 (s, 2H), 3.84 (s, 3H), 3.71 (s, 6H), 3.43 (s, 3H), 2.07–2.04 (m, 9H), 1.75–1.72 (m, 6H). ^13^C NMR (100 MHz, DMSO-d_6_) δ 193.9, 162.2, 158.4, 158.1, 145.0, 138.6, 127.9, 127.8, 127.7, 127.4, 115.1, 111.7, 94.2, 91.5, 56.6, 56.2, 55.9, 40.5, 37.1, 36.9, 28.8. ESI-MS: *m*/*z* calcd. for C_30_H_37_O_6_^+^ [M + H]^+^ 493.26, found 493.35.

*4-((E)-3-(3-adamantan-1-yl)-4-(methoxymethoxy)phenyl)acryloyl)-3-hydroxybenzoic acid (**CA-13**)*

A yellow solid, yield: 30.4%. mp 196−198 °C. IR (KBr, cm^−1^): 2910, 2852 (aliphatic C–H, adamantyl); 1640 (C=O); 1572 (C=C, conjugated & Ar); 1492 (Ar C=C/C–H); 1133 (C–O–C, ether); 983 (trans-CH=CH out-of-plane bending). ^1^H NMR (400 MHz, CHLOROFORM-d) δ 11.26 (s, 1H), 8.52 (d, *J* = 2.3 Hz, 1H), 8.10 (dd, *J* = 2.2, 8.7 Hz, 1H), 7.73 (d, *J* = 15.7 Hz, 1H), 7.47 (d, *J* = 2.1 Hz, 1H), 7.41 (dd, *J* = 2.1, 8.6 Hz, 1H), 7.33 (d, *J* = 15.5 Hz, 1H), 7.07 (d, *J* = 8.4 Hz, 1H), 7.01 (d, *J* = 8.8 Hz, 1H), 5.21 (s, 2H), 3.46 (s, 3H), 2.09–2.02 (m, 9H), 1.75–1.70 (m, 6H). ^13^C NMR (100 MHz, CHLOROFORM-d) δ 188.1, 169.9, 165.1, 158.6, 145.3, 139.1, 135.6, 131.4, 130.1, 128.1, 127.7, 127.5, 119.1, 117.9, 114.7, 112.5, 94.1, 56.4, 40.6, 37.2, 37.1, 29.0. ESI-MS: *m*/*z* calcd. for C_28_H_29_O_6_^−^ [M − H]^−^ 461.20, found 461.20.

### 2.2. Cell Culture

Human lung cancer cell lines A549 (ATCC^®^ CCL-185), H460 (ATCC^®^ HTB-177), H292 (ATCC^®^ CRL-1481), and the normal lung fibroblast line MRC-5 (ATCC^®^ CCL-171) were obtained from the American Type Culture Collection (ATCC) (Manassas, VA, USA). A549, H460, and H292 cells were maintained in Dulbecco’s Modified Eagle Medium (DMEM) supplemented with 10% fetal bovine serum (FBS) and 1% penicillin–streptomycin. MRC-5 cells were cultured in Minimum Essential Medium (MEM) containing 10% FBS. All cells were incubated at 37 °C in a humidified atmosphere with 5% CO_2_.

### 2.3. Cell Viability Assay (MTT Assay)

Cell viability was determined using the MTT assay. Briefly, H460 and H292 cells were seeded into 96-well plates at a density of 1 × 10^4^ cells per well and treated with various concentrations of the test compounds (2.5, 5, 10, 20, 30, 40, 50 and 80 µM) for 24 h. For mechanistic studies, cells were pretreated with 3-methyladenine (3-MA, 10 mM, 2 h), chloroquine (CQ, 3 µM, 1 h), or SP600125 (10 µM, 2 h) prior to CA13 exposure for an additional 24 h. Subsequently, 20 µL of MTT solution (5 mg/mL) was added to each well and incubated for 4 h at 37 °C. The supernatant was carefully removed, and the resulting formazan crystals were dissolved in 150 µL DMSO. Absorbance was measured at 490 nm using a microplate reader (PerkinElmer, Waltham, MA, USA). The density of formazan formed in the blank group was set as 100% of viability. Cell viability (%) = [sample (OD_490_)/blank (OD_490_)] × 100%. Blank group: cultured with fresh medium only. Sample group: treated with compounds. All experiments were performed in triplicate.

### 2.4. Western Blot Analysis

Western blotting was performed as previously described [30]. The following primary antibodies were used: anti-PARP (ab227244), LC3B (ab192890), ATG7 (ab52472), pJNK (ab124956), JNK (ab124956), β-actin (ab6276), and GAPDH (ab181602) (Abcam, Cambridge, UK). Total protein was extracted from cells using RIPA lysis buffer, and protein concentration was quantified with a BCA Protein Assay Kit (P0010, Beyotime Biotechnology, Shanghai, China). Protein samples were separated by SDS-PAGE and transferred onto PVDF membranes (Millipore, IPVH00010, Billerica, MA, USA). The membranes were blocked with 5% non-fat milk in TBST (pH 7.6) for 1.5 h at room temperature, followed by incubation with primary antibodies overnight at 4 °C. After three washes with TBST, the membranes were incubated with HRP-conjugated goat anti-mouse (1:5000, Abclonal, Hangzhou, China) or goat anti-rabbit (1:5000, Abclonal) secondary antibodies for 1 h at room temperature. Protein bands were visualized using an enhanced chemiluminescence kit (Advansta, Inc., San Jose, CA, USA) according to the manufacturer’s instructions. Original figures can be found in Supplementary Materials. 

### 2.5. Flow Cytometry Analysis of Apoptosis

Annexin V-FITC/PI apoptosis detection was performed as previously described [30]. Briefly, H292 cells were seeded in 6-well plates. After adherence, cells were treated with CA13 (0, 2.5, 5 and 10 µM) for 24 h, harvested using trypsin (without EDTA) at 37 °C, and washed twice with cold PBS. Cell pellets were resuspended in 250 µL of 1 × binding buffer, followed by staining with 5 µL Annexin V-FITC and 5 µL propidium iodide (PI) (Yeasen, Inc., Shanghai, China) for 15 min at room temperature in the dark. Apoptotic cell populations were analyzed using a flow cytometer (Beckman Coulter, CA, USA). Data analysis was conducted with FlowJo software (version 10.8.1), distinguishing early apoptotic (Annexin V+/PI−), late apoptotic (Annexin V+/PI+), and necrotic (Annexin V−/PI+) cells.

### 2.6. Autophagic Flux Analysis

mRFP-GFP-LC3 plasmids (Hanbio, Nanjing, China) were used to transfect H292 cells according to the manufacturer’s instructions, and were then treated with CA13 (2.5 or 5 μM) for 24 h. The formation of autolysosomes was observed under a Leica TCS SP8 STED confocal laser scanning microscope (Leica Microsystems GmbH, Wetzlar, Germany), and images were captured at 400× magnification.

### 2.7. Xenograft Mouse Model

All animal experiments were approved by the Institutional Animal Care and Use Committee of Hubei University of Science and Technology. Five-week-old male BALB/c-nu/nu mice were maintained under specific pathogen-free conditions. H292 cells (1 × 10^6^) suspended in 100 µL PBS were injected subcutaneously into the right flank of each mouse. When tumor volume reached approximately 100 mm^3^, mice were randomized into three groups (*n* = 5 per group) and administered PBS (vehicle control), CA13 at 20 mg/kg, or CA13 at 40 mg/kg by intraperitoneal injection every other day. Tumor volume and body weight were measured every three days for 24 days.

### 2.8. Statistical Analysis

Data are expressed as means ± standard deviation (SD) from three independent experiments. Comparisons between two groups were assessed using Student’s two-tailed unpaired *t*-test. Statistical analysis was performed using GraphPad Prism 8.0. A *p*-value < 0.05 was considered statistically significant.

## 3. Results

### 3.1. Chemistry

**Scheme 1:**
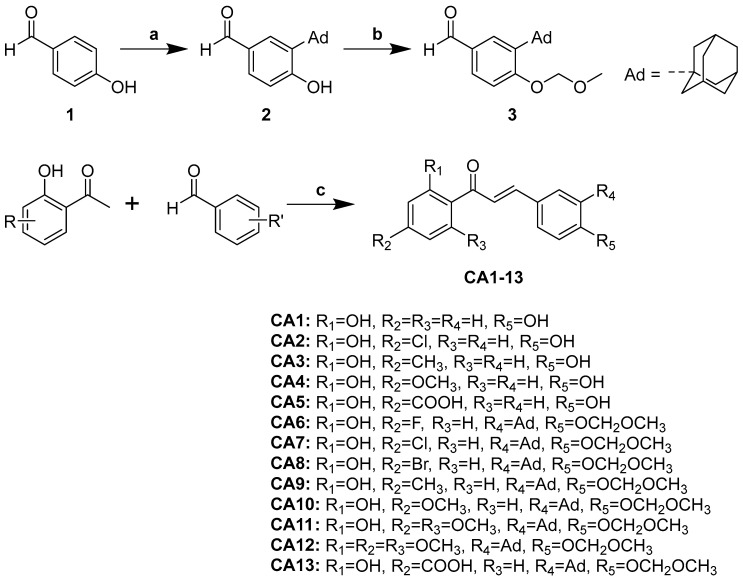
Synthetic route of chalcones **CA 1–13**. Reagents and conditions: (a) H_2_SO_4,_ acetic acid, 70 °C, 6 h; (b) K_2_CO_3_, MOMCl, acetone, 60 °C, 12 h; (c) NaOH, MeOH, 70 °C, 12–24 h.

Chalcones **CA 1–13** were synthesized via aldol condensation of corresponding aldehydes (1 mmol) and various acetophenones (1 mmol) in methanolic basic medium as shown in Scheme 1. The characterization of **CA 1–4** was reported previously [31,32,33]. Friedel-Crafts alkylation of 4-hydroxybenzaldehyde yielded 3-adamanty-l-2- hydroxybenzaldehyde, which was then treated with chloromethyl methyl ether (MOMCl) to afford the adamantyl-substituted hydroxybenzaldehyde (**2**), following our reported procedure [30]. Aldol condensation of relevant acetophenones with the adamantyl-substituted hydroxybenzaldehyde, performed with NaOH in MeOH at 70 °C, provided new adamantyl-substituted chalcones **CA 6–13**. Structures were confirmed by melting points, NMR (^1^H and ^13^C NMR), and mass spectrometry.

### 3.2. Biological Activity

#### 3.2.1. In Vitro Antiproliferative Activity of Adamantly-Substituted Chalcones on Lung Cancer Cell Lines

To explore the antitumor activity of these chalcone derivatives, all compounds were first assayed for in vitro antiproliferative activities against H292 and A549 cancer cell lines using an MTT assay with 5-fluorouracil (5-FU) included as a positive control. The concentrations of compounds required for 50% inhibition of cell viability (IC_50_) were determined and summarized in Table 1. The results indicated that more than half of the synthesized chalcone derivatives exhibited antiproliferative activity against both H292 and A549 cells with IC_50_ values below 50 µM. Among them, five compounds (**CA7**, **CA8**, **CA10**, **CA11**, and **CA13**) were active toward H292 cells (IC_50_ < 35 µM), and three (**CA7**, **CA11**, and **CA13**) also exhibited good inhibitory activity against A549 cells (IC_50_ < 30 µM). Notably, compound **CA13** showed the most potent cytotoxic effect against both tested cell lines (IC_50_ = 10.15 µM for H292 and 12.24 µM for A549), significantly lower than those of the reference compounds 2′-HC (IC_50_ > 50 μM for both cell lines) and 5-FU (28.83 ± 2.31 μM for H292 and 21.56 ± 2.04 μM for A549). The results summarized in Table 1 suggest that different substituents on the phenyl rings (ring-A and ring-B) have remarkable influences on antiproliferative potency.

Using OH substituents at R1 and R5, we examined the antiproliferative activities of **CA1–CA5** having different groups at R2 position (H, Cl, CH_3_, OCH_3_, and COOH, respectively). Among them, compound **CA5** with a COOH group at R2 exhibited the strongest antiproliferative activity against both cancer cell lines (IC_50_ = 38.17 μM for H292 and 41.35 μM for A549), followed by **CA2** with a Cl substituent. In contrast, compounds with electron-donating groups, such as CH_3_ (**CA3**) or OCH_3_ (**CA4**), showed weaker or no activity (IC_50_ > 45 μM). These results indicate that the introduction of electron-withdrawing substituents (COOH, Cl) at the R2 position of ring-A enhances the antiproliferative effect. Further comparison between **CA2–CA5** (R4 = H) and **CA7–CA10** (R4 = Ad, R5 = OCH_2_OCH_3_) revealed that incorporation of the bulky adamantyl group at ring-B significantly improved antiproliferative potency. For example, **CA7** (R2 = Cl) and **CA8** (R2 = Br) displayed much lower IC_50_ values (22.24 and 21.57 μM against H292, respectively) than their non-adamantyl analogues **CA2** and **CA4**. This enhancement suggests that the adamantyl group might contribute to increased lipophilicity and better cellular uptake [30,34], thereby enhancing cytotoxic efficacy.

In addition, **CA11**, bearing dual methoxy groups at R2 and R3, exhibited improved activity (IC_50_ = 21.06 μM for H292 and 24.03 μM for A549) compared with its mono-methoxy analogue **CA10** (34.18 μM and 32.16 μM, respectively), indicating that multiple electron-donating groups can synergistically enhance antiproliferative potency when combined with the adamantyl moiety. Remarkably, **CA13**, which carries both a COOH group at R2 and an adamantyl substituent at R4, showed the best antiproliferative activity among all tested compounds (IC_50_ = 10.15 μM for H292 and 12.24 μM for A549), while its non-adamantyl analogue **CA5** was much less active. This observation highlights a synergistic effect between the hydrophilic carboxyl group and the bulky hydrophobic adamantyl moiety, which appears to be critical for high cytotoxic potency.

Next, the most active compound, **CA13**, was selected for further evaluation of its antiproliferative activity against the additional lung cancer cell line (H460) and a normal human lung fibroblast line (MRC-5). As shown in Table 2, **CA13** exhibited broad-spectrum cytotoxicity against A549, H460, and H292 cancer cells with IC_50_ values ranging from 10.15 to 12.24 µM. Notably, **CA13** showed negligible toxicity toward normal MRC-5 cells (IC_50_ > 50 µM), whereas the positive control 5-FU exhibited measurable toxicity (IC_50_ = 40.72 ± 2.26 μM) and the reference 2′-HC exhibited weak or no activity. These results highlight **CA13** as a promising lead compound with both potent and selective antiproliferative activity, warranting further investigation.

#### 3.2.2. Adamantly-Substituted Chalcone **CA13** Induced Autophagy in Lung Cancer Cells

Autophagy, a type II programmed cell death mechanism, plays a critical role in maintaining cellular homeostasis and modulating cancer cell fate under therapeutic stress. Among the core regulators of autophagy, microtubule-associated protein 1 light chain 3 (LC3) is essential for the biogenesis of autophagosomes and is widely recognized as a molecular hallmark of autophagic activation. During autophagy initiation, cytosolic LC3-I undergoes conjugation with phosphatidylethanolamine to form LC3-II through an Atg7/Atg3-mediated ubiquitin-like system, enabling autophagosomal membrane elongation and closure [27,35].

**Figure 2:**
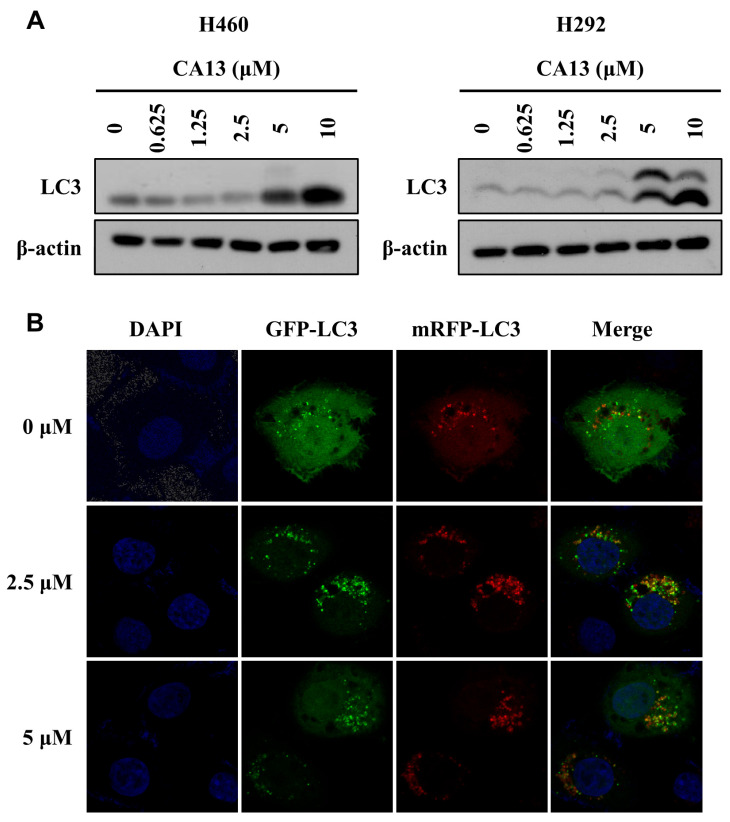
**CA13** induced autophagy and promoted autophagy flux in lung cancer cells. (**A**) Western blot analyses of LC-3 in cells treated with **CA13** for 24 h. β-actin was used as an internal control. Original blots can be found in Supplementary Materials. (**B**) The H292 cells were transfected with a plasmid encoding mRFP-GFP-LC3 and treated with **CA13** for 24 h. Images were captured with confocal microscopy. Scale bar: 10 µm.

Previous studies have reported that 2′-hydroxychalcones are capable of inducing autophagy in lung cancer cells [24]. Based on these findings, we explored whether adamantyl-substituted chalcones exhibit similar autophagy-inducing properties. Western blot analysis revealed a concentration-dependent increase in LC3A/B-II expression following **CA13** treatment (Figure 2A), suggesting enhanced autophagosome formation. To further verify that **CA13** activates complete autophagic flux rather than impeding autophagosome degradation, a tandem fluorescent mRFP-GFP-LC3 reporter assay was employed. As shown in Figure 2B, **CA13** treatment markedly increased the number of LC3 puncta in a concentration-dependent manner. The predominance of red-only puncta indicated efficient fusion between autophagosomes and lysosomes, demonstrating that **CA13** not only stimulates autophagosome formation but also facilitates their maturation into autolysosomes. Together, these findings indicate that **CA13** acts as a potent inducer of autophagy in H292 cells, facilitating both the initiation of autophagosome formation and the progression of autophagic flux.

#### 3.2.3. **CA13** Induced Apoptosis in Lung Cancer Cells

**Figure 3:**
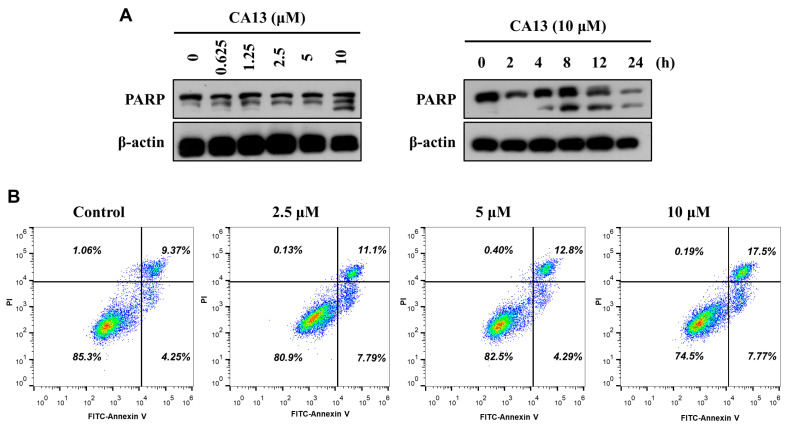
**CA13** induces apoptosis in H292 lung cancer cells. (**A**) Western blot analysis of cleaved PARP expression in H292 cells treated with various concentrations of **CA13** (0–10 μM) for 24 h or with 10 μM **CA13** for the indicated time periods. β-actin was used as a loading control. Original blots can be found in Supplementary Materials. (**B**) H292 cells were treated with **CA13** at the indicated concentrations for 24 h and analyzed by Annexin V FITC/PI staining. Apoptotic cell ratio was quantified by flow cytometry. Data are presented as mean ± standard deviation (SD) for three independent experiments.

Apoptosis has been widely reported as a key mechanism of chalcone-induced cytotoxicity in various cancer cell lines [8]. To determine whether apoptosis contributes to the cytotoxic effects of **CA13** in lung cancer cells, we first examined the expression of apoptosis-related proteins. Western blot analysis (Figure 3A) demonstrated that **CA13** treatment increased the levels of cleaved PARP, a hallmark of apoptosis, in both a dose- and time-dependent manner. To further confirm the pro-apoptotic effect of **CA13**, Annexin V-FITC/PI double staining followed by flow cytometric analysis was performed in H292 cells. Annexin V binds to phosphatidylserine residues exposed on the outer leaflet of the plasma membrane, allowing discrimination between viable, early apoptotic, and late apoptotic cells when combined with PI staining. As shown in the representative flow cytometry plots (Figure 3B), **CA13** treatment significantly and dose-dependently increased the proportion of apoptotic cells. The percentage of late apoptotic cells rose from 11.1 ± 1.7% at 2.5 μM to 17.5 ± 2.4% at 10 μM after 12 h of treatment. These results indicate that **CA13** induces apoptotic cell death in H292 lung cancer cells.

#### 3.2.4. **CA13**-Induced Autophagy Plays a Protective Role in Lung Cancer Cells

**Figure 4:**
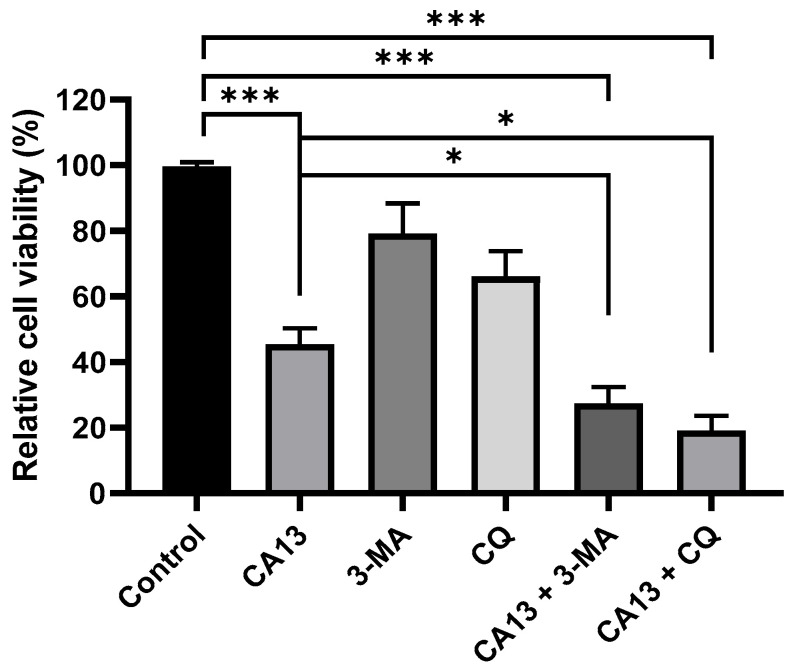
**WA13** induces cytoprotective autophagy in H292 cells. Cell viability of H292 cells was measured after treatment with **CA13** (10 μM), 3-methyladenine (3-MA, 10 mM), chloroquine (CQ, 3 μM), or their combination for 24h using MTT assays. Both early (3-MA) and late (CQ) autophagy inhibitors significantly reduced cell viability when combined with **CA13**, suggesting that inhibition of autophagy enhances **CA13**-induced cytotoxicity. Data are presented as mean ± standard deviation (SD) for three independent experiments. * *p* < 0.05, *** *p* < 0.001.

Accumulating evidence indicates that autophagy and apoptosis are closely interconnected in cancer cells, with autophagy exhibiting either cytoprotective or cytotoxic effects depending on the cellular context and tumor microenvironment [36,37]. To elucidate the role of **CA13**-induced autophagy in H292 cells, inhibitors targeting different stages of autophagy were employed. As shown in Figure 4, inhibition of autophagy by either 3-MA or CQ significantly enhanced CA13-induced cell death, as demonstrated by decreased cell viability in MTT assays. These results suggest that **CA13**-induced autophagy plays a protective role in H292 cells by attenuating apoptotic cell death.

#### 3.2.5. JNK Activation Is Required for **CA13**-Induced Apoptosis in Lung Cancer Cells

**Figure 5:**
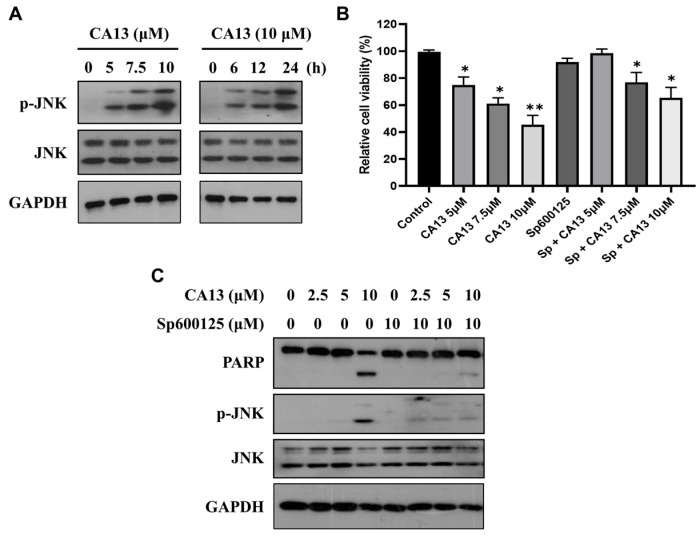
JNK activation is required for **CA13**-induced apoptosis in H292 cells. (**A**) H292 cells were treated with **CA13** (0–10 μM) for 24 h or with 10 μM **CA13** for the indicated time periods. The expression of JNK and p-JNK was analyzed by Western blotting, with GAPDH as a loading control. (**B**) Cell viability of H292 cells was measured using MTT assays after treatment with **CA13** (10 μM), SP600125 (10 μM), or their combination for 24 h. (**C**) H292 cells were pretreated with SP600125 (10 μM) for 6 h, followed by incubation with **CA13** (10 μM) for 24 h. The protein levels of PARP, JNK, and p-JNK were determined by Western blotting. Original blots can be found in Supplementary Materials. Data are presented as mean ± standard deviation (SD) for three independent experiments. * *p* < 0.05, ** *p* < 0.01.

Activation of the c-Jun N-terminal kinase (JNK) signaling pathway has been widely implicated in chalcone-mediated apoptosis across various cancer cell types [38,39,40,41,42]. To determine whether **CA13**-induced apoptosis depends on JNK activation, we first examined JNK phosphorylation in H292 cells. As shown in Figure 5A, **CA13** treatment markedly increased the phosphorylation level of JNK (p-JNK) in both dose- and time-dependent manners, while total JNK levels remained unchanged. To further validate the role of JNK activation in **CA13**-induced cytotoxicity, the specific JNK inhibitor SP600125 was applied. Pretreatment with SP600125 significantly restored cell viability compared with **CA13** treatment alone (Figure 5B). Western blot analysis confirmed that SP600125 effectively blocked JNK phosphorylation and attenuated **CA13**-induced cleavage of PARP (Figure 5C). These results demonstrate that JNK activation is essential for CA13-induced apoptosis in H292 cells, highlighting the critical role of the JNK signaling pathway in mediating the pro-apoptotic effects of **CA13**.

#### 3.2.6. **CA13** Suppresses Lung Cancer Growth In Vivo

**Figure 6:**
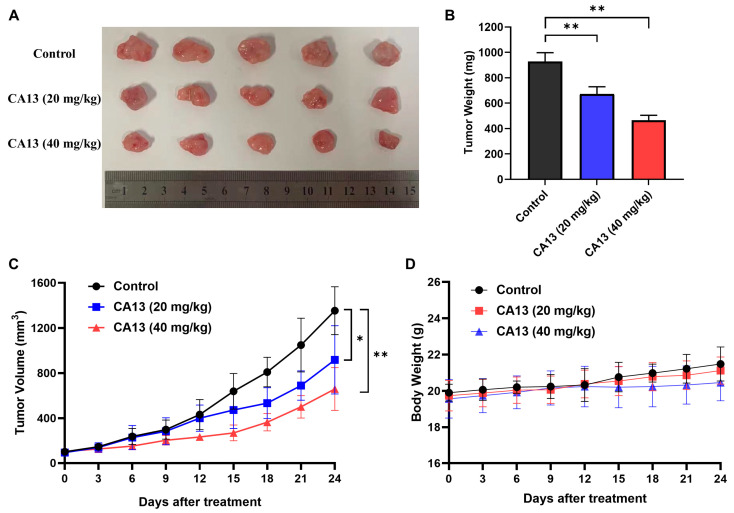
**CA13** suppresses tumor growth in the H292 xenograft mouse model. (**A**,**B**) Tumor-bearing Balb/c nude mice (*n* = 5 per group) were treated with vehicle or **CA13** (20 or 40 mg/kg) via intraperitoneal injection every three days. Tumor volume was measured at the indicated time points. (**C**) Tumors were excised and weighed after 24 days of treatment. (**D**) Body weight was monitored throughout the experimental period to evaluate potential systemic toxicity. Data are presented as mean ± standard deviation (SD) for three independent experiments. * *p* < 0.05, ** *p* < 0.01.

To evaluate the antitumor efficacy of **CA13** in vivo, a xenograft model was established by subcutaneous implantation of H292 cells into Balb/c nude mice. Once tumors became palpable, mice were intraperitoneally administered **CA13** at doses of 20 or 40 mg/kg, or vehicle control, every three days. As shown in Figure 6A,B, **CA13** treatment significantly inhibited tumor growth in a dose-dependent manner compared with the control group (*p* < 0.05). Final tumor weight analysis confirmed the pronounced reduction in tumor burden in **CA13**-treated mice (Figure 6C). Importantly, no significant difference in body weight was observed among the groups throughout the treatment period (Figure 6D), suggesting that **CA13** did not cause overt systemic toxicity. These findings demonstrate that **CA13** exhibits potent antitumor activity against human H292 lung cancer xenografts in vivo, while maintaining a favorable safety profile.

## 4. Discussion

In this study, a series of adamantyl-substituted chalcones were synthesized and evaluated for their anticancer properties. Among them, **CA13** exhibited the most potent antiproliferative activity against human lung cancer cells while showing minimal cytotoxicity toward normal lung fibroblasts. Structural–activity analysis suggested that the introduction of an adamantyl group and a carboxyl substituent synergistically enhanced cytotoxic potency, likely by improving molecular lipophilicity and π–π conjugation, consistent with prior studies reporting enhanced bioactivity through hydrophobic modification of chalcones [30,34]. Notably, our previous work identified WA15, a structurally analogous adamantyl-substituted chalcone that shares the core adamantyl and carboxyl pharmacophores with CA13 and exhibits comparable in vitro anti-NSCLC potency [30]. Nonetheless, the mechanistic focuses of the two studies are fundamentally distinct. The prior investigation of WA15 established its role as an RXRα modulator, demonstrating its ability to inhibit RXRα transactivation, induce proteasome-dependent RXRα degradation [30]. In contrast, the present study on CA13 centers on elucidating how CA13-induced autophagy regulates lung cancer cell apoptosis. Specifically, we confirm a cytoprotective role of autophagy against CA13-triggered apoptotic stress, a previously unexplored axis.

Mechanistic analyses revealed that **CA13** triggers both autophagy and apoptosis in H292 lung cancer cells. Western blot and fluorescence microscopy demonstrated significant LC3-II accumulation and formation of autolysosomes, confirming activation of complete autophagic flux. Interestingly, inhibition of autophagy potentiated **CA13**-induced apoptosis, suggesting that autophagy serves a cytoprotective role under **CA13**-induced stress. This duality of autophagy, functioning either as a survival or death mechanism, has been widely observed in tumor responses to chemotherapeutic chalcones [21,22,23,24,25,26].

Consistent with previous studies on chalcone analogues, **CA13** triggered apoptotic cell death in lung cancer cells and JNK activation is indispensable for CA13-induced apoptosis. Similar JNK-dependent apoptotic mechanisms have been described for other chalcones, such as xanthohumol and butein, where sustained JNK activation promotes mitochondrial dysfunction and caspase activation [39,40,41]. Our results reinforce that JNK signaling acts as a central mediator of **CA13**-induced apoptosis in lung cancer cells.

The interplay between autophagy and apoptosis represents a critical determinant of therapeutic response. While **CA13** concurrently activated both processes, functional studies revealed that autophagy mitigates **CA13**-induced apoptotic stress, functioning as a survival mechanism. Therefore, dual targeting of autophagy and apoptosis could represent a rational strategy to potentiate **CA13**’s anticancer activity.

## 5. Conclusions

In summary, **CA13** is a novel adamantyl chalcone derivative that exerts potent cytotoxic effects through JNK-dependent apoptosis and cytoprotective autophagy. These findings not only highlight **CA13** as a promising therapeutic lead against lung cancer but also provide mechanistic insights into how structural modification of chalcones can modulate the balance between survival and death pathways. Future studies will focus on identifying upstream molecular targets of **CA13** and evaluating combination therapies that inhibit autophagy to enhance its antitumor efficacy.

## Figures and Tables

**Table 1:** In vitro antiproliferative activities of target compounds against cancer H292 cells.

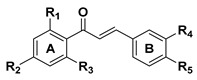							
Comp.	R1	R2	R3	R4	R5	IC_50_ (μmol/L) ^1^	
						H292	A549
2′-HC	OH	H	H	H	H	>50	>50
CA1	OH	H	H	H	OH	>50	>50
CA2	OH	Cl	H	H	OH	40.56 ± 2.16	48.04 ± 2.01
CA3	OH	CH_3_	H	H	OH	>50	>50
CA4	OH	OCH_3_	H	H	OH	45.25 ± 1.85	>50
CA5	OH	COOH	H	H	OH	38.17 ± 2.35	41.35 ± 2.13
CA6	OH	F	H	Ad	OCH_2_OCH_3_	>50	>50
CA7	OH	Cl	H	Ad	OCH_2_OCH_3_	22.24 ± 1.80	25.46 ± 1.21
CA8	OH	Br	H	Ad	OCH_2_OCH_3_	21.57 ± 2.17	27.32 ± 1.74
CA9	OH	CH_3_	H	Ad	OCH_2_OCH_3_	>50	>50
CA10	OH	OCH_3_	H	Ad	OCH_2_OCH_3_	34.18 ± 2.09	32.16 ± 2.28
CA11	OH	OCH_3_	OCH_3_	Ad	OCH_2_OCH_3_	21.06 ± 1.57	24.03 ± 1.82
CA12	OCH3	OCH_3_	OCH_3_	Ad	OCH_2_OCH_3_	40.31 ± 2.04	43.52 ± 2.24
CA13	OH	COOH	H	Ad	OCH_2_OCH_3_	10.15 ± 1.61	12.24 ± 1.28
5-FU	-	-	-	-	-	28.83 ± 2.31	21.56 ± 2.04

^1^ Data are means ± SD of triplicate experiments.

**Table 2:** In vitro antiproliferative activities of selected compounds against cancer and normal cells.

Comp.	IC_50_ (μmol/L) ^1^			
	A549	H460	H292	MRC-5
CA13	12.24 ± 1.28	10.60 ± 0.67	10.15 ± 1.61	>50
2′-HC	>50	46.83 ± 2.15	>50	>50
5-FU	21.56 ± 2.04	27.57 ± 1.92	28.83 ± 2.31	40.72 ± 2.26

^1^ Data are means ± SD of triplicate experiments.

## Data Availability

The data is available via the attached Supplementary Materials provided above.

## Supplementary Materials

The following supporting information can be downloaded at: https://www.mdpi.com/article/10.3390/biom16010054/s1, ^1^H NMR and ^13^C NMR data for compounds **CA6–CA13**. Figure S1. ^1^H NMR Spectrum of compound CA 6. Figure S2. ^13^C NMR Spectrum of compound CA 6. Figure S3. Mass Spectrum of compound CA 6. Figure S4. IR Spectrum of compound CA 6. Figure S5. ^1^H NMR Spectrum of compound CA 7. Figure S6. ^13^C NMR Spectrum of compound CA 7. Figure S7. Mass Spectrum of compound CA 7. Figure S8. IR Spectrum of compound CA 7. Figure S9. ^1^H NMR Spectrum of compound CA 8. Figure S10. ^13^C NMR Spectrum of compound CA 8. Figure S11. Mass Spectrum of compound CA 8. Figure S12. IR Spectrum of compound CA 8. Figure S13. ^1^H NMR Spectrum of compound CA 9. Figure S14. ^13^C NMR Spectrum of compound CA 9. Figure S15. Mass Spectrum of compound CA 9. Figure S16. IR Spectrum of compound CA 9. Figure S17. ^1^H NMR Spectrum of compound CA 10. Figure S18. ^13^C NMR Spectra of compound CA 10. Figure S19. Mass Spectrum of compound CA 10. Figure S20. IR Spectrum of compound CA 10. Figure S21. ^1^H NMR Spectrum of compound CA 11. Figure S22. ^13^C NMR Spectra of compound CA 11. Figure S23. Mass Spectrum of compound CA 11. Figure S24. IR Spectrum of compound CA 11. Figure S25. ^1^H NMR Spectra of compound CA 12. Figure S26. ^13^C NMR Spectra of compound CA 12. Figure S27. Mass Spectrum of compound CA 12. Figure S28. IR Spectrum of compound CA 12. Figure S29. ^1^H NMR Spectra of compound CA 13. Figure S30. ^13^C NMR Spectra of compound CA 13. Figure S31. Mass Spectrum of compound CA 13. Figure S32. IR Spectrum of compound CA 13. Original Western blots.
