# Supplementary material for: Adamantyl-Substituted Chalcone CA13 Induces Cytoprotective Autophagy and JNK-Dependent Apoptosis in Lung Cancer Cells
Source: Biomolecules. 2025 Dec 30;16(1):54. doi: 10.3390/biom16010054 (PMC12838717; doi:10.3390/biom16010054)
Supplement: Supplementary file 1 [file biomolecules-16-00054-s001.zip › biomolecules-4007400-supplementary.pdf]

# Adamantyl-Substituted Chalcone CA13 Induces Cytoprotective Autophagy and JNK-Dependent Apoptosis in Lung Cancer Cells

**Yuting Chen [1,†], Yaxin Liu [1,†], Jing Zhou [1], Tingting Bao [1], Jing Wang [2,*] and Mingtao Ao [1,3,*]**

[1]School of Pharmacy, Hubei University of Science and Technology, Xianning 437100, China

[2]Institutes of Biomedical Science, Inner Mongolia University, Hohhot 010021, China

[3]Xiantao First People's Hospital, The Affiliated Hospital of Hubei University of Science and Technology, Xiantao 433000, China

*Correspondence: jingwang@imu.edu.cn (J.W.); aomingtao@hbust.edu.cn (M.A.)

[†] These authors contributed equally to this work.

# Content


Figure S1. $^{1}H$ NMR Spectrum of compound CA 6........................................................3
Figure S2. $^{13}C$ NMR Spectrum of compound CA 6.......................................................3
Figure S3. Mass Spectrum of compound CA 6..............................................................4
Figure S4. IR Spectrum of compound CA 6 ...................................................................4
Figure S5. $^{1}H$ NMR Spectrum of compound CA 7........................................................5
Figure S6. $^{13}C$ NMR Spectrum of compound CA 7.......................................................5
Figure S7. Mass Spectrum of compound CA 7..............................................................6
Figure S8. IR Spectrum of compound CA 7 ...................................................................6
Figure S9. $^{1}H$ NMR Spectrum of compound CA 8........................................................7
Figure S10. $^{13}C$ NMR Spectrum of compound CA 8.....................................................7
Figure S11. Mass Spectrum of compound CA 8............................................................8
Figure S12. IR Spectrum of compound CA 8 .................................................................8
Figure S13. $^{1}H$ NMR Spectrum of compound CA 9......................................................9
Figure S14. $^{13}C$ NMR Spectrum of compound CA 9.....................................................9
Figure S15. Mass Spectrum of compound CA 9..........................................................10
Figure S16. IR Spectrum of compound CA 9 ...............................................................10
Figure S17. $^{1}H$ NMR Spectrum of compound CA 10..................................................11
Figure S18. $^{13}C$ NMR Spectra of compound CA 10 ....................................................11
Figure S19. Mass Spectrum of compound CA 10........................................................12
Figure S20. IR Spectrum of compound CA 10 ............................................................12
Figure S21. $^{1}H$ NMR Spectrum of compound CA 11 ..................................................13
Figure S22. $^{13}C$ NMR Spectra of compound CA 11 ....................................................13
Figure S23. Mass Spectrum of compound CA 11........................................................14
Figure S24. IR Spectrum of compound CA 11 ............................................................14
Figure S25. $^{1}H$ NMR Spectra of compound CA 12 .....................................................15
Figure S26. $^{13}C$ NMR Spectra of compound CA 12....................................................15
Figure S27. Mass Spectrum of compound CA 12........................................................16
Figure S28. IR Spectrum of compound CA 12 ............................................................16
Figure S29. $^{1}H$ NMR Spectra of compound CA 13 .....................................................17
Figure S30. $^{13}C$ NMR Spectra of compound CA 13....................................................17
Figure S31. Mass Spectrum of compound CA 13........................................................18
Figure S32. IR Spectrum of compound CA 13 ............................................................18
Original Western blots.........................................................................................19

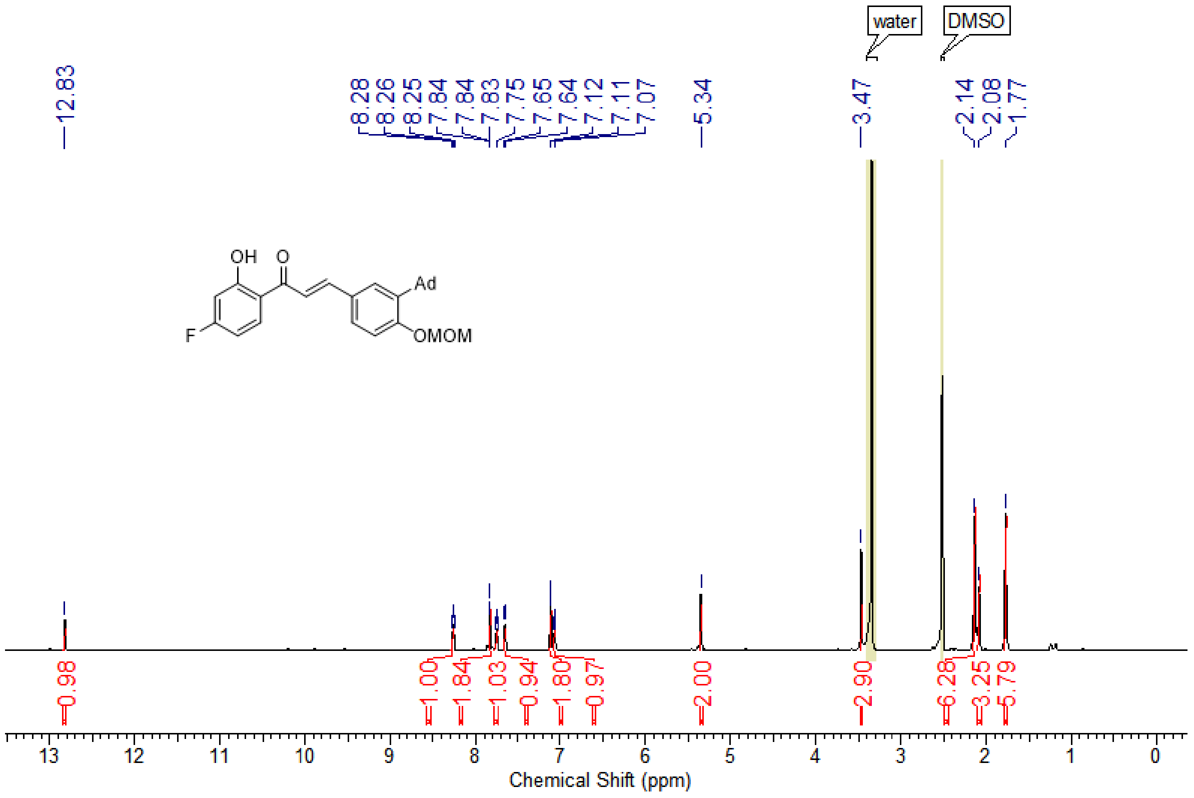


**Figure S1**. $^{1}$H NMR Spectrum of compound CA 6

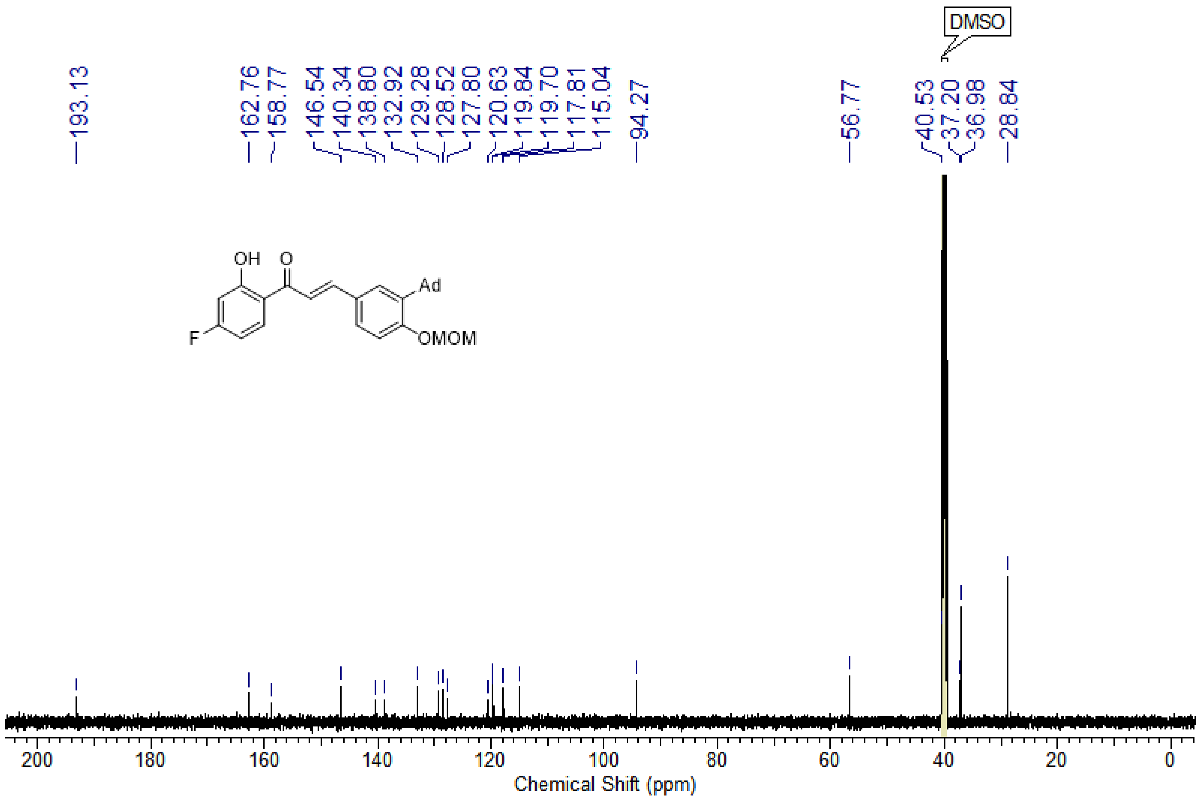


**Figure S2.** $^{13}$C NMR Spectrum of compound **CA 6**

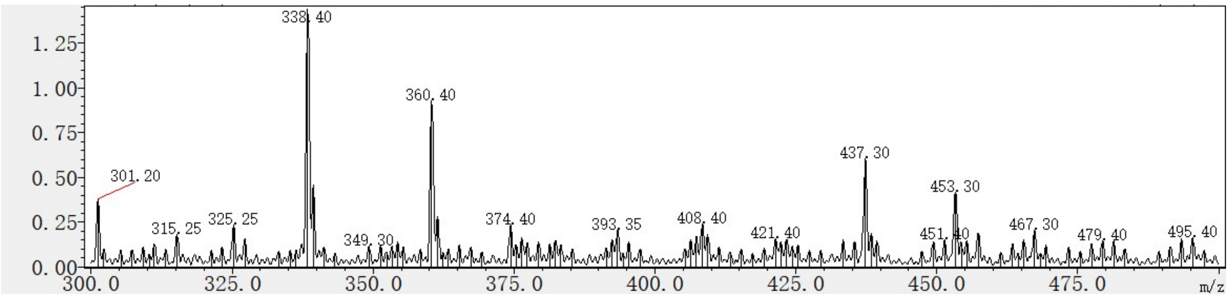


**Figure S3.** Mass Spectrum of compound **CA 6**

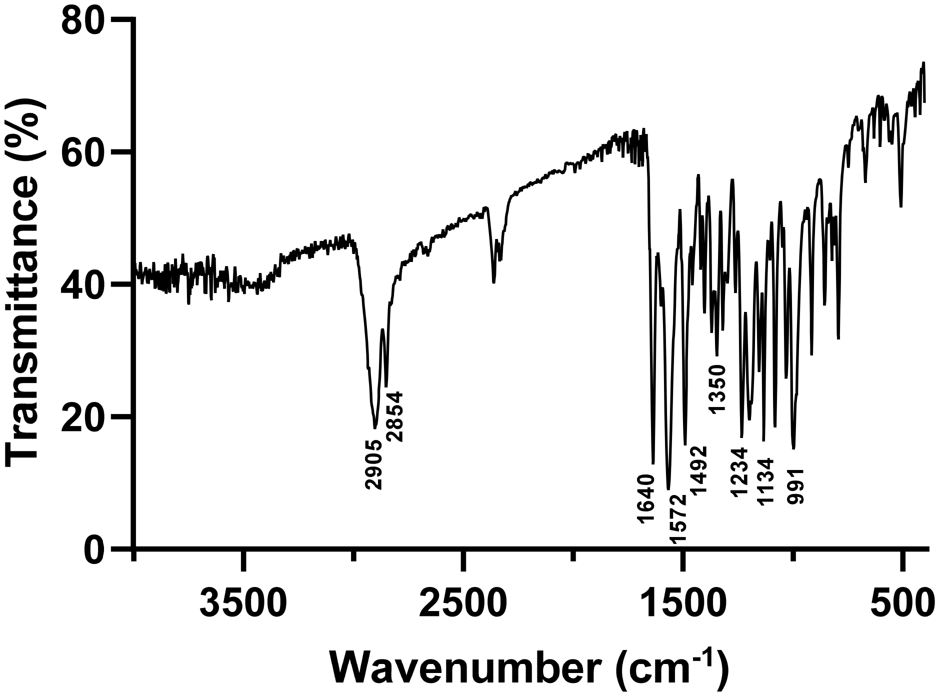


**Figure S4.** IR Spectrum of compound **CA 6**

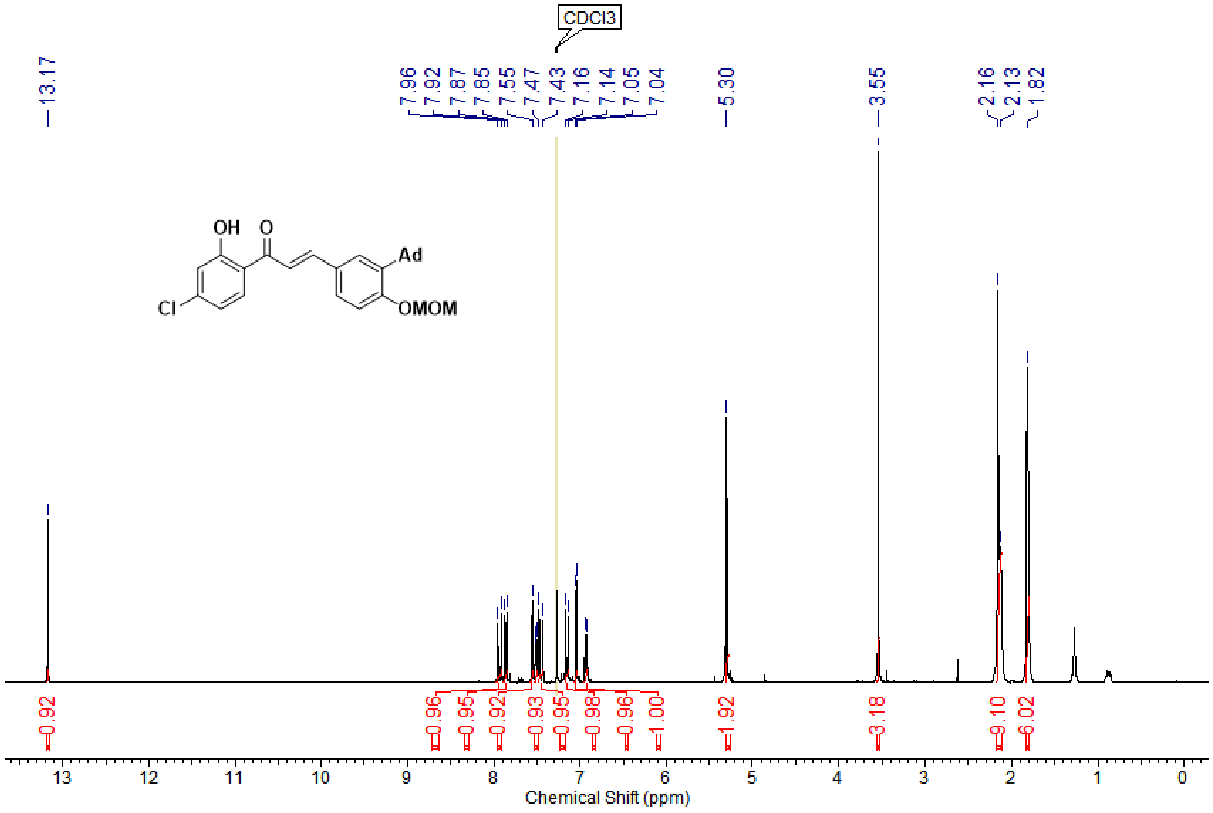

**Figure S5.** $^{1}H$ NMR Spectrum of compound **CA 7**

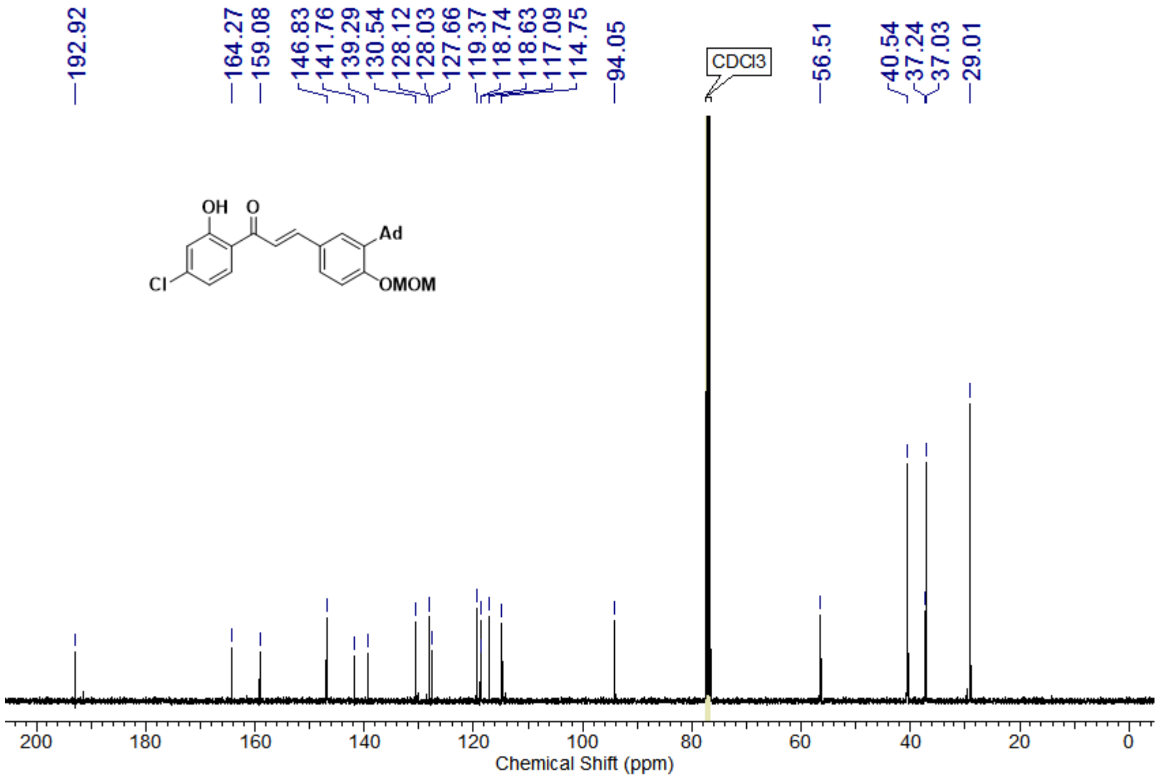

**Figure S6.** $^{13}C$ NMR Spectrum of compound **CA 7**

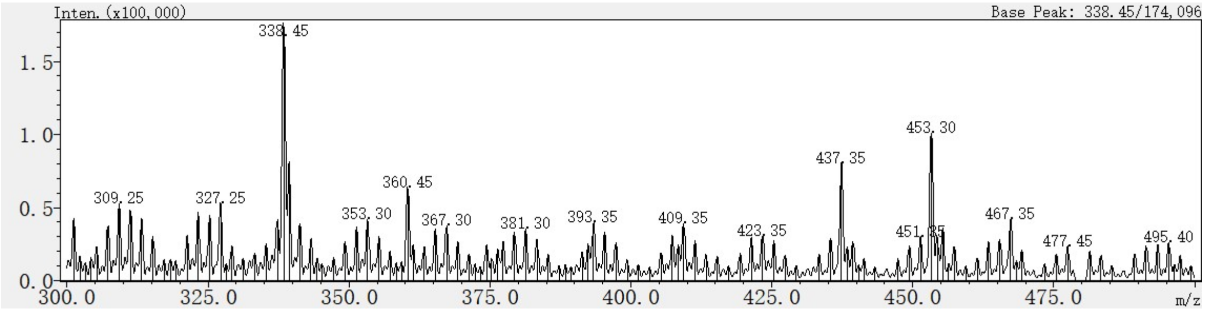


**Figure S7**. Mass Spectrum of compound **CA 7**

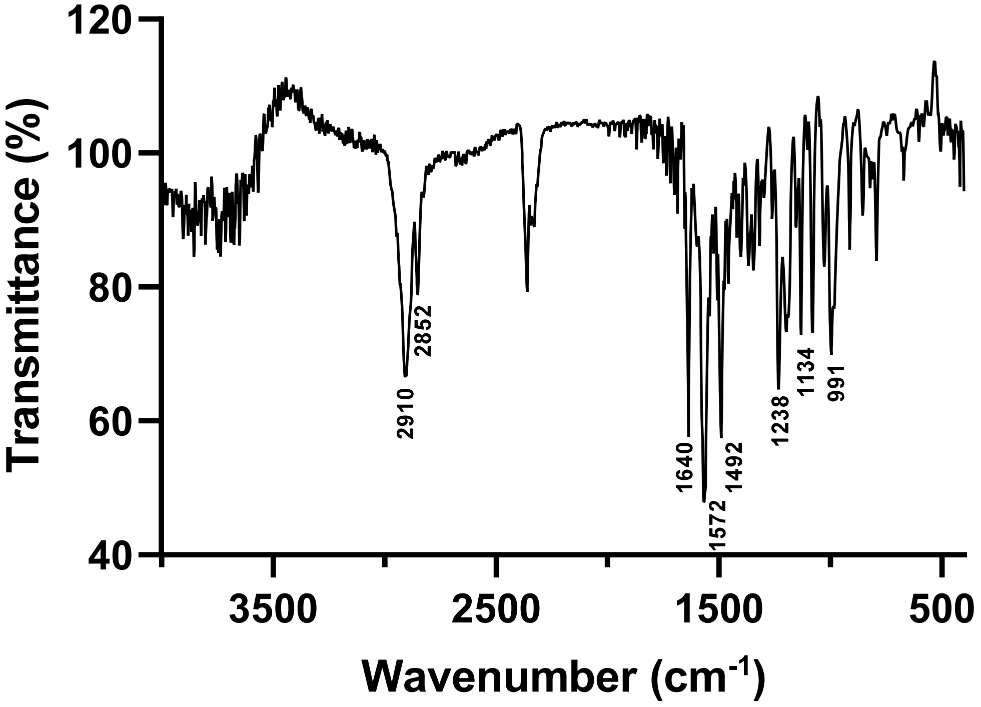


**Figure S8.** IR Spectrum of compound **CA 7**

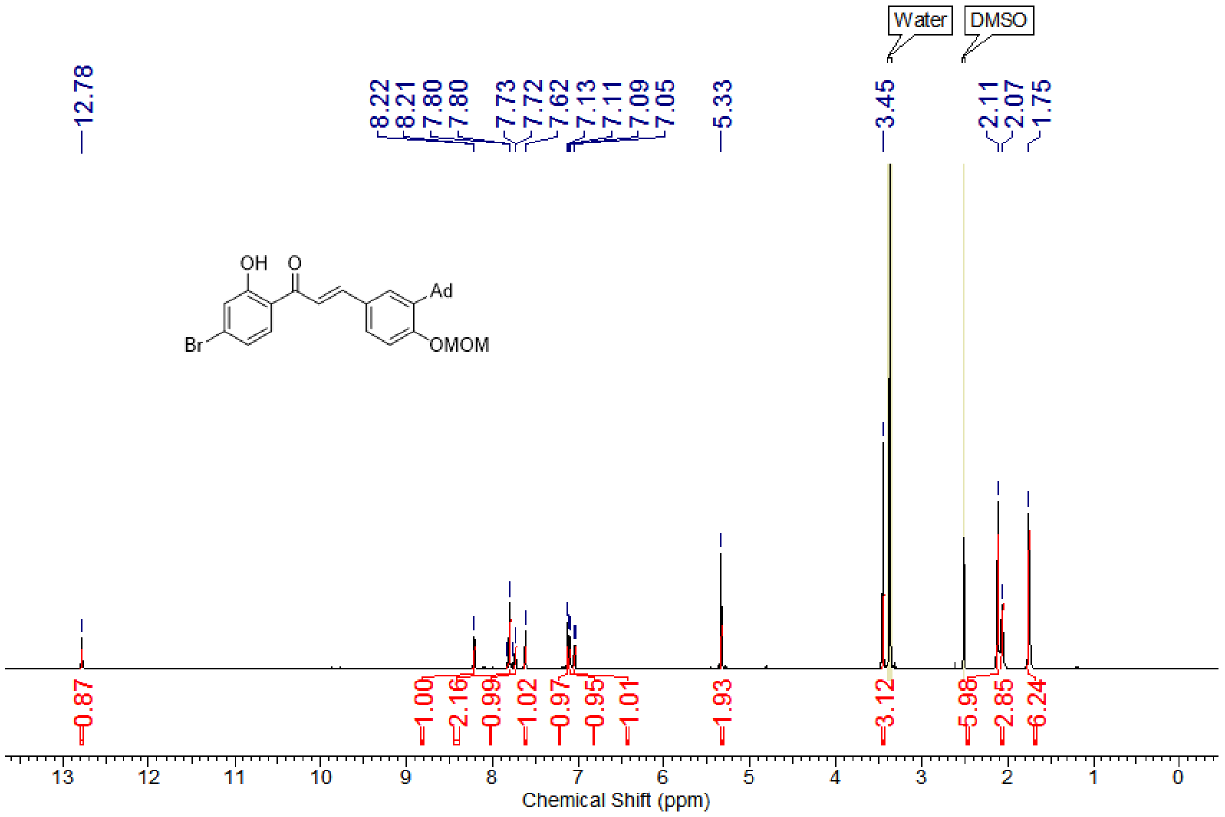


**Figure S9.** $^{1}$H NMR Spectrum of compound **CA 8**

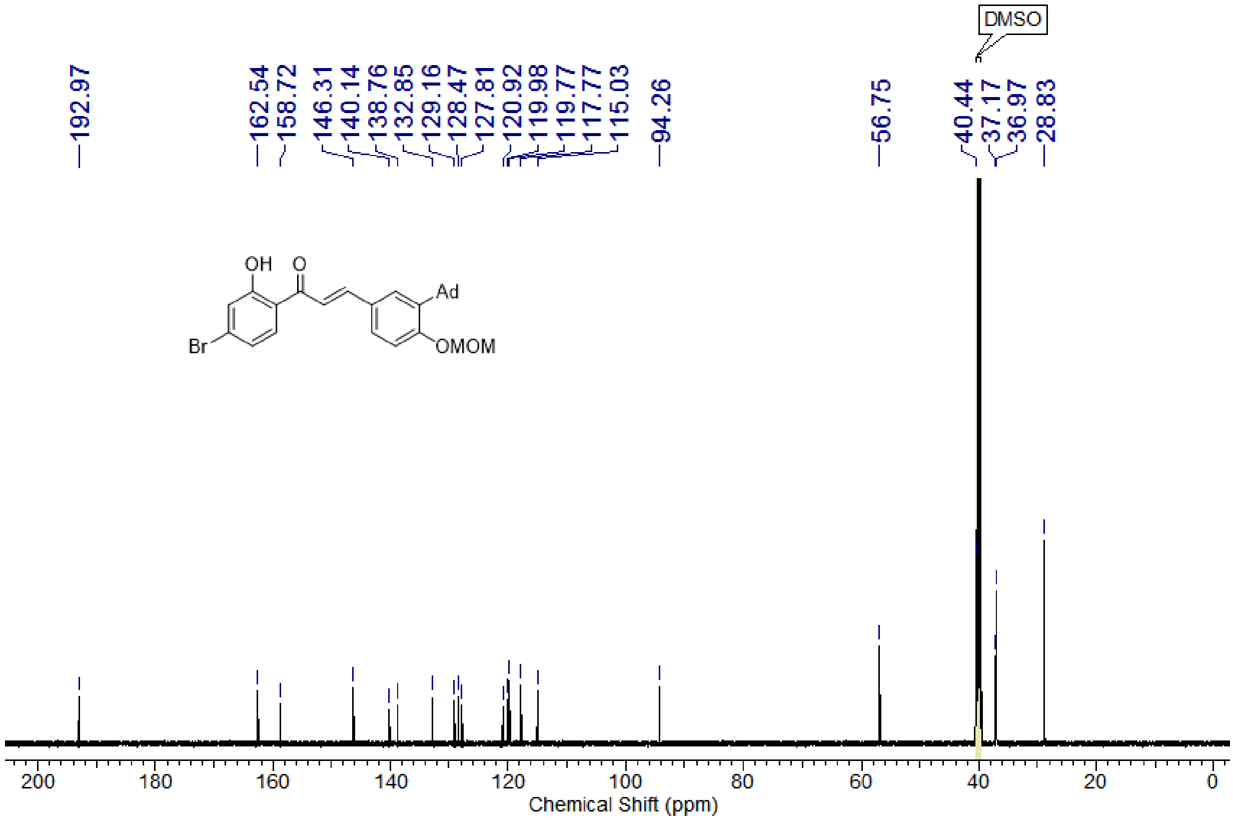


**Figure S10.** $^{13}$C NMR Spectrum of compound **CA 8**

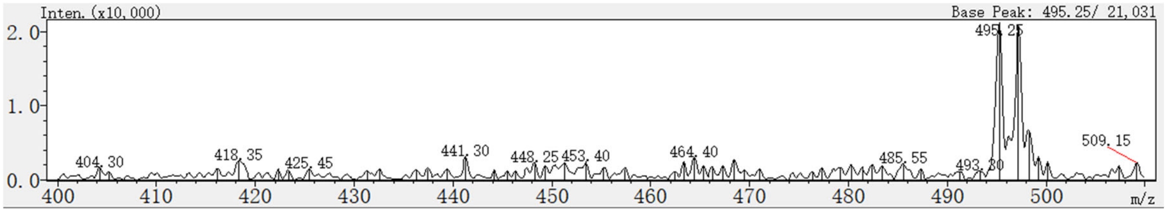


**Figure S11**. Mass Spectrum of compound **CA 8**

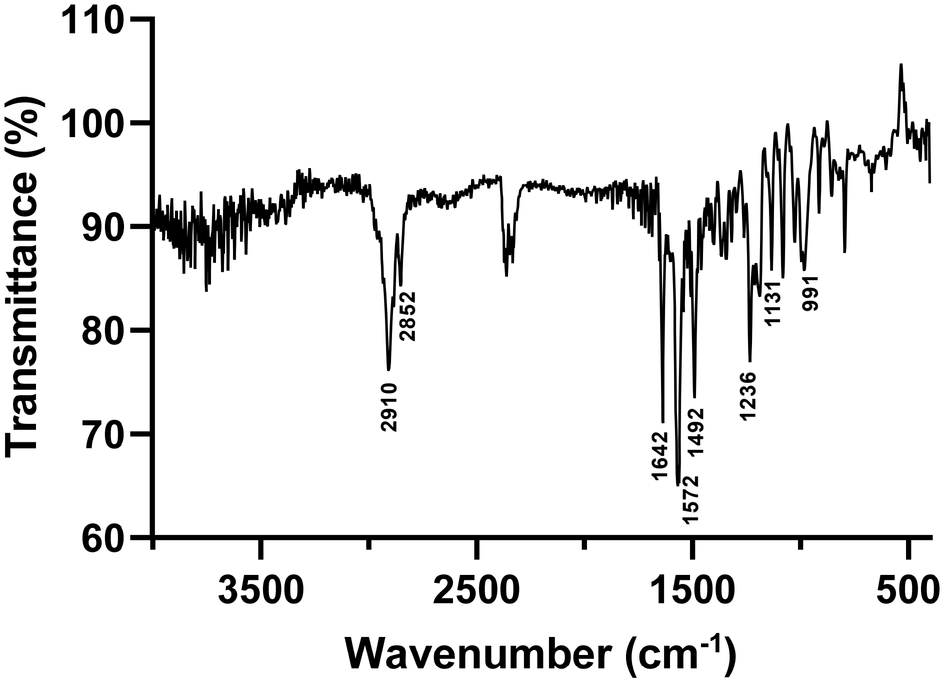


**Figure S12.** IR Spectrum of compound **CA 8**

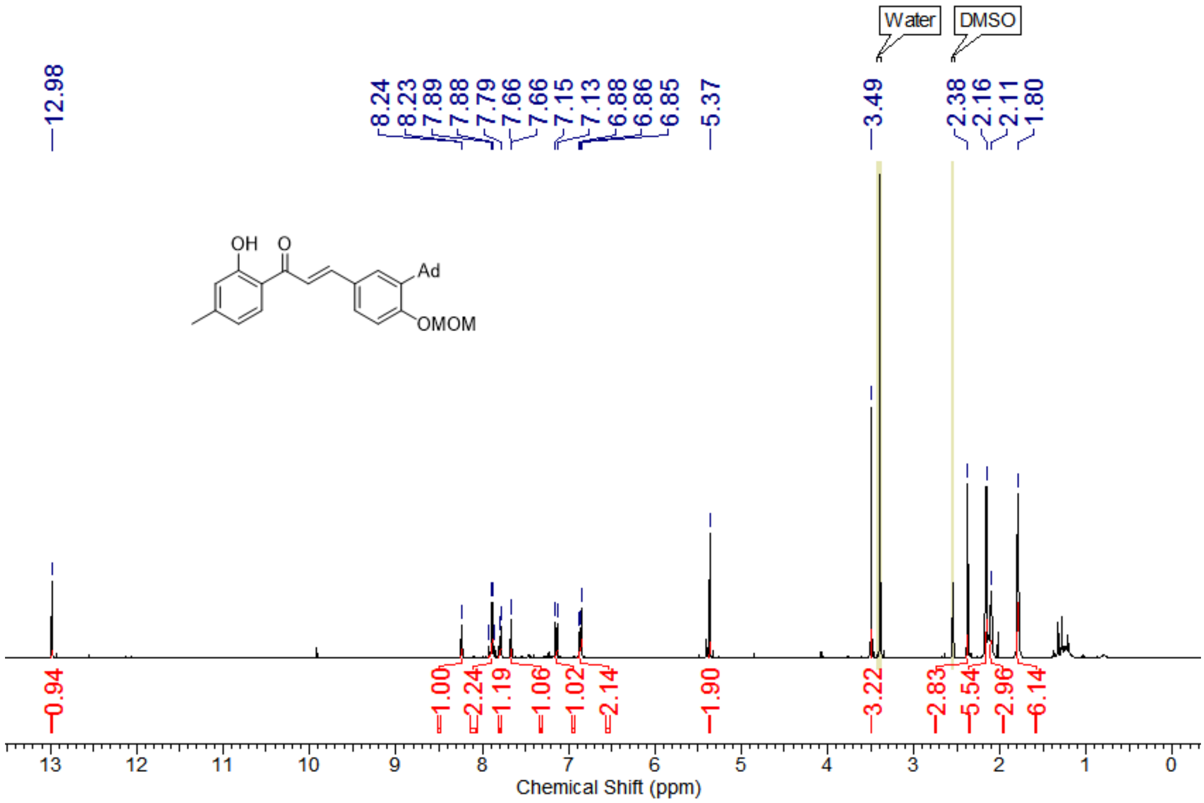


**Figure S13.** $^{1}$H NMR Spectrum of compound **CA 9**

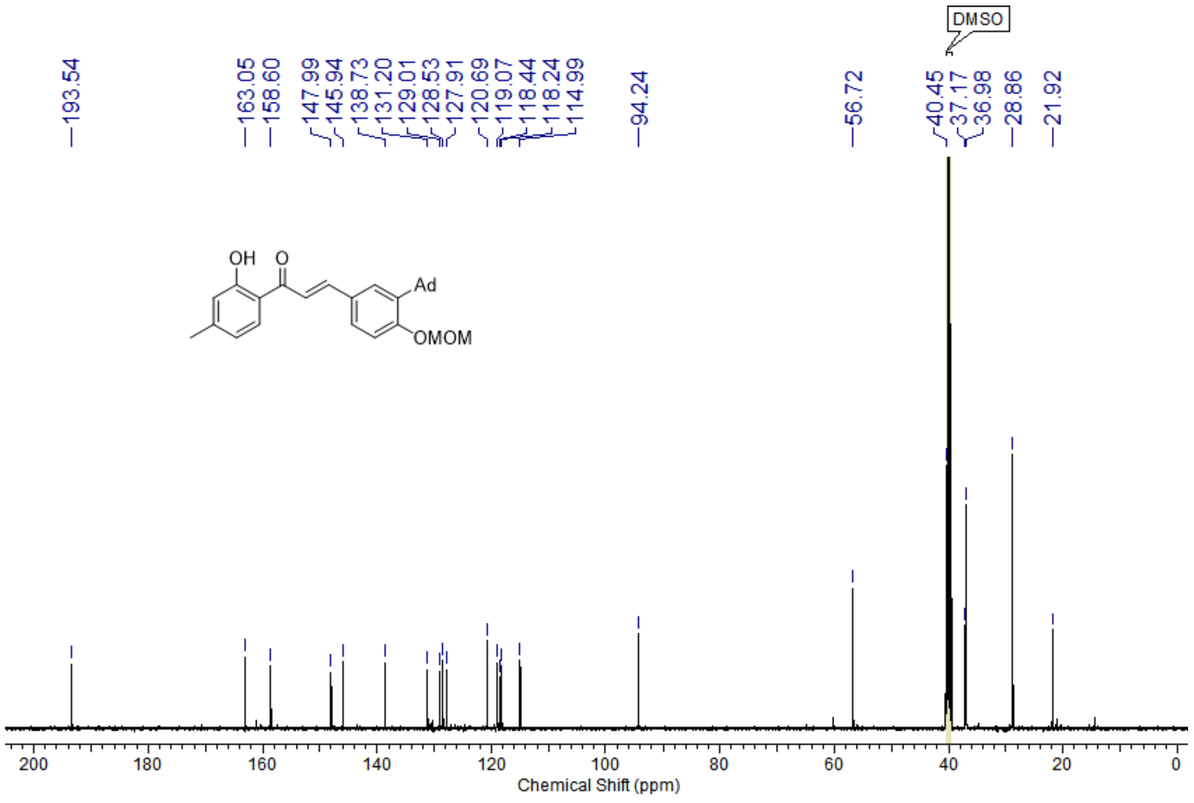


**Figure S14.** $^{13}$C NMR Spectrum of compound **CA 9**

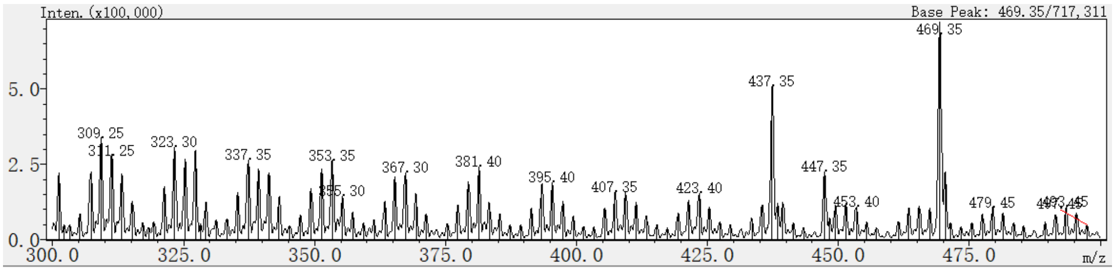


**Figure S15.** Mass Spectrum of compound **CA 9**

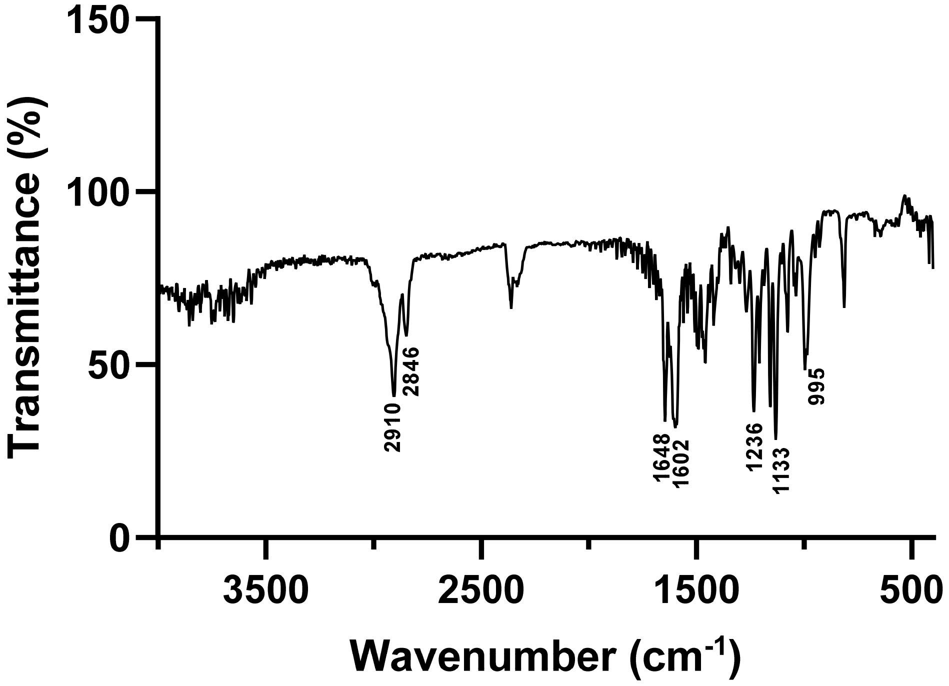


**Figure S16.** IR Spectrum of compound **CA 9**

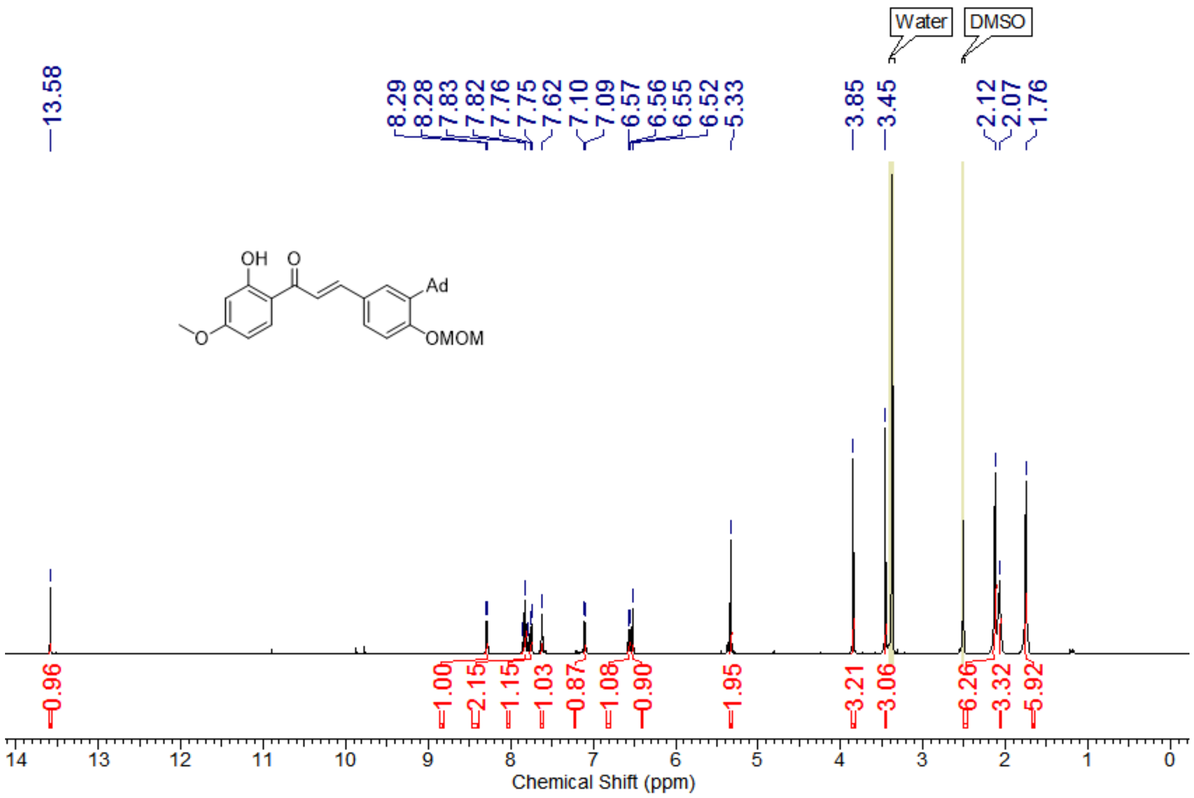


**Figure S17.** $^{1}$H NMR Spectrum of compound **CA 10**

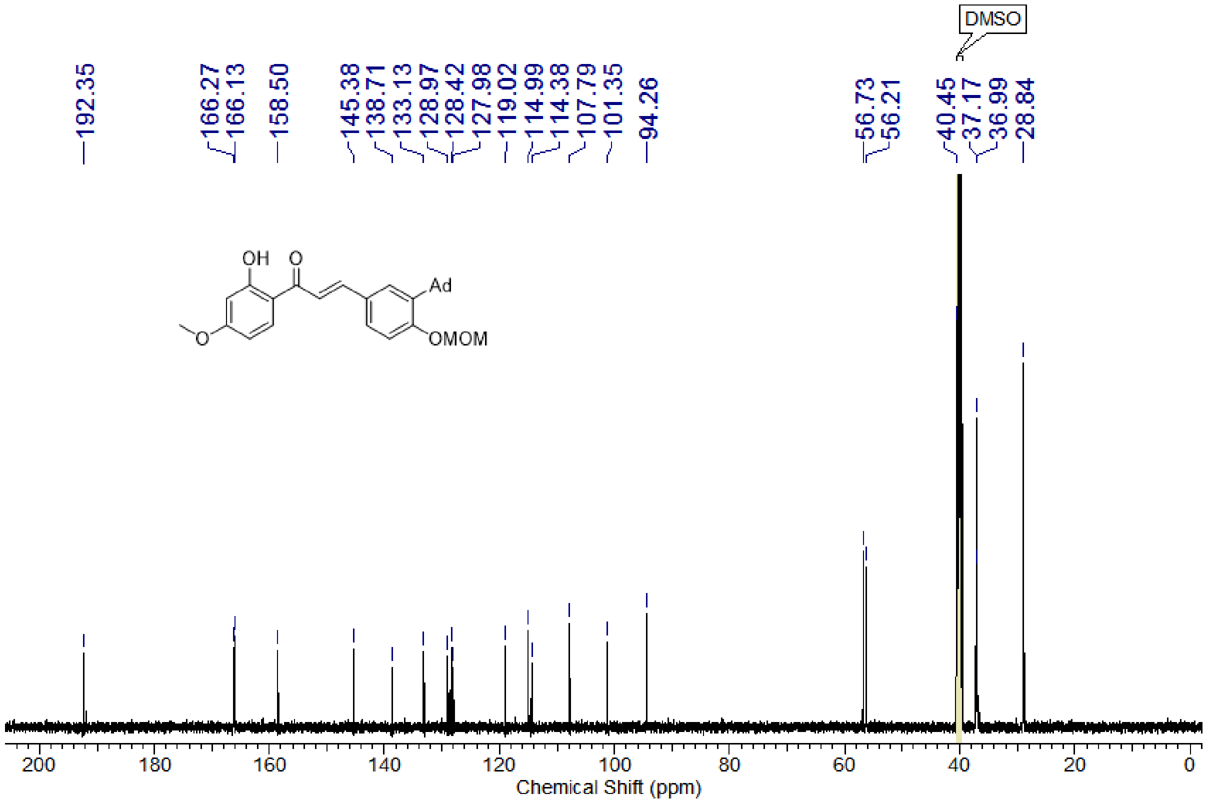


**Figure S18.** $^{13}$C NMR Spectra of compound **CA 10**

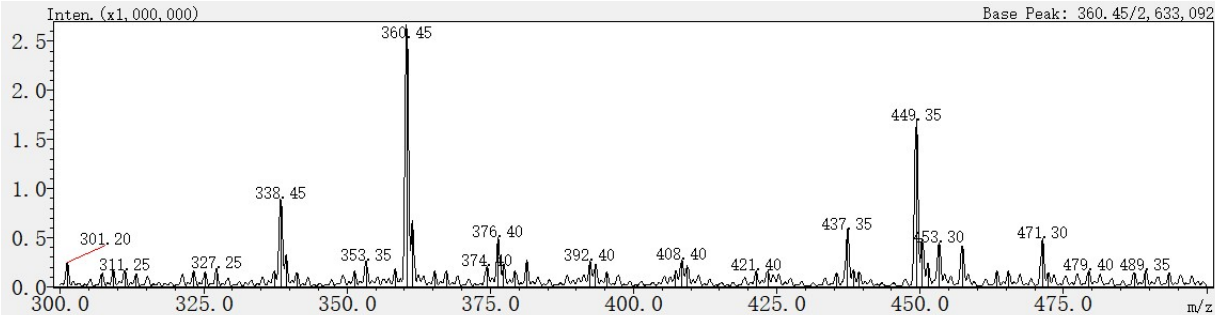


**Figure S19.** Mass Spectrum of compound **CA 10**

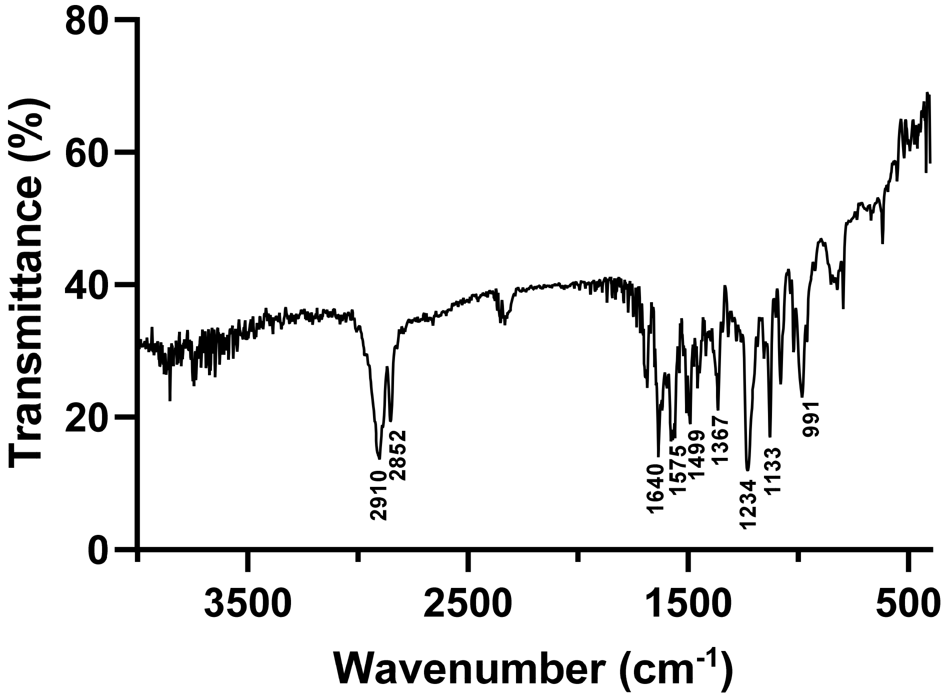


**Figure S20.** IR Spectrum of compound **CA 10**

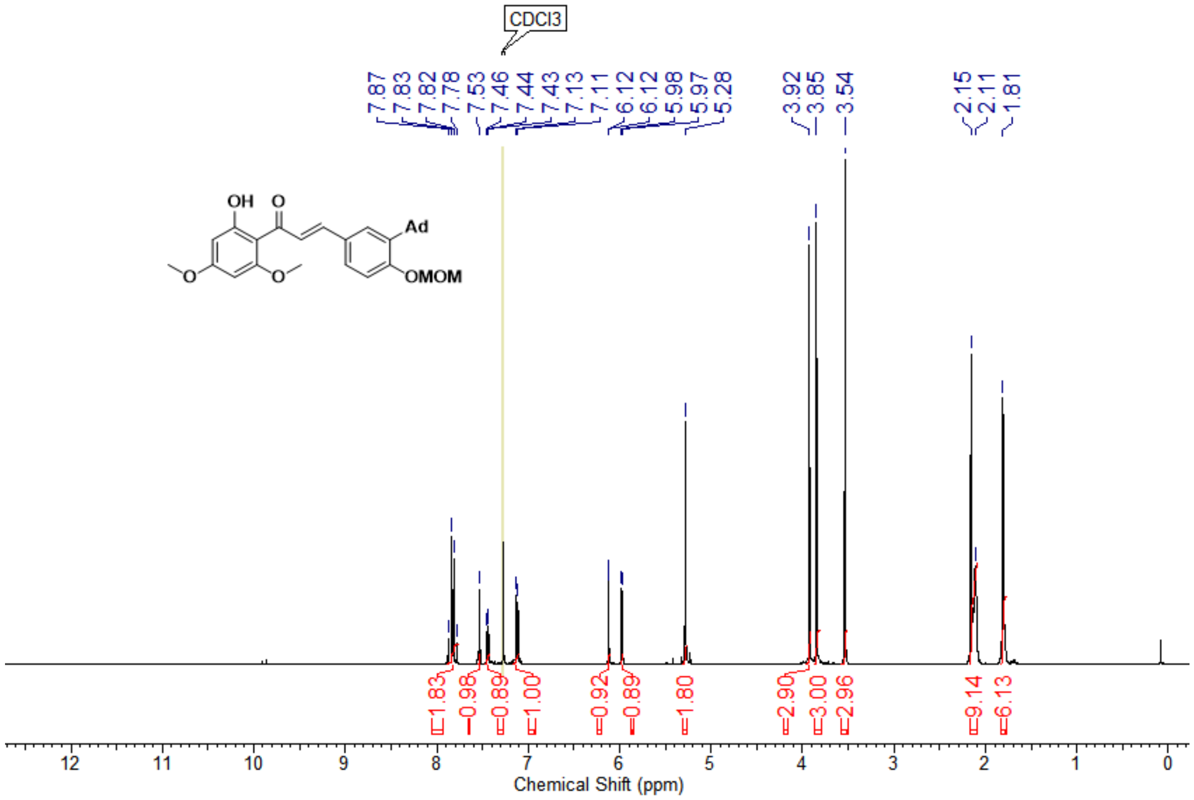


**Figure S21.** $^{1}$H NMR Spectrum of compound **CA 11**

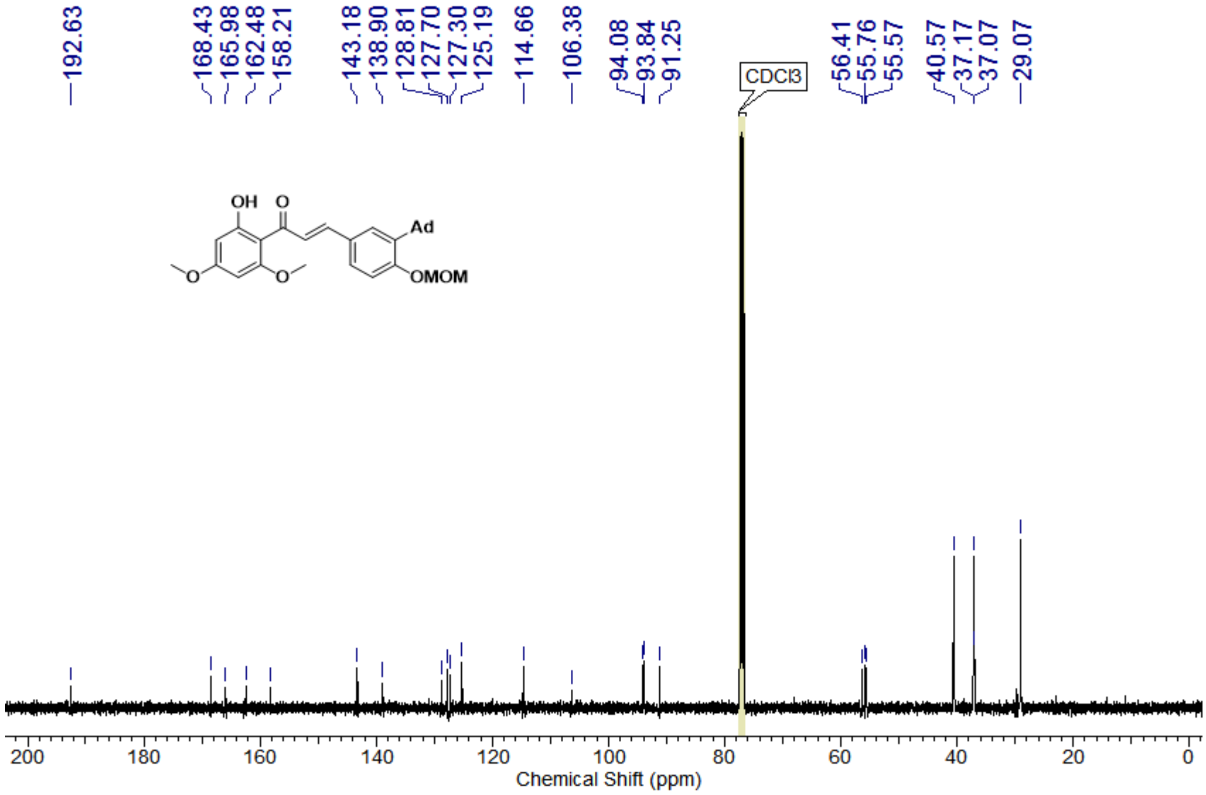


**Figure S22.** $^{13}$C NMR Spectra of compound **CA 11**

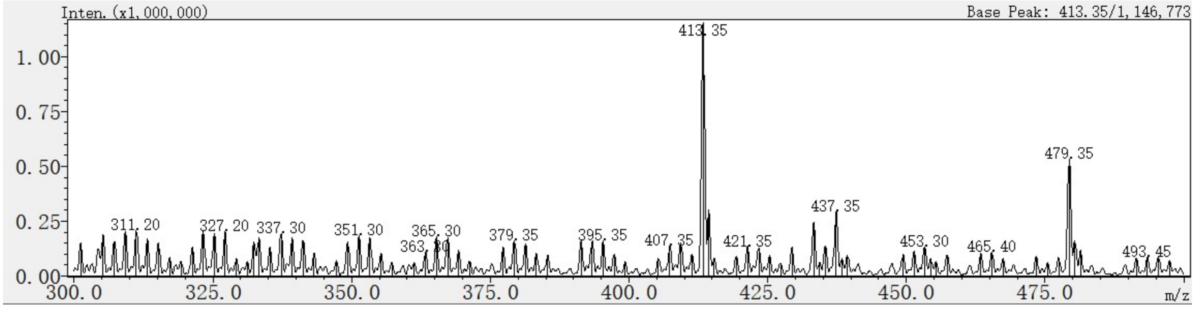


**Figure S23.** Mass Spectrum of compound **CA 11**

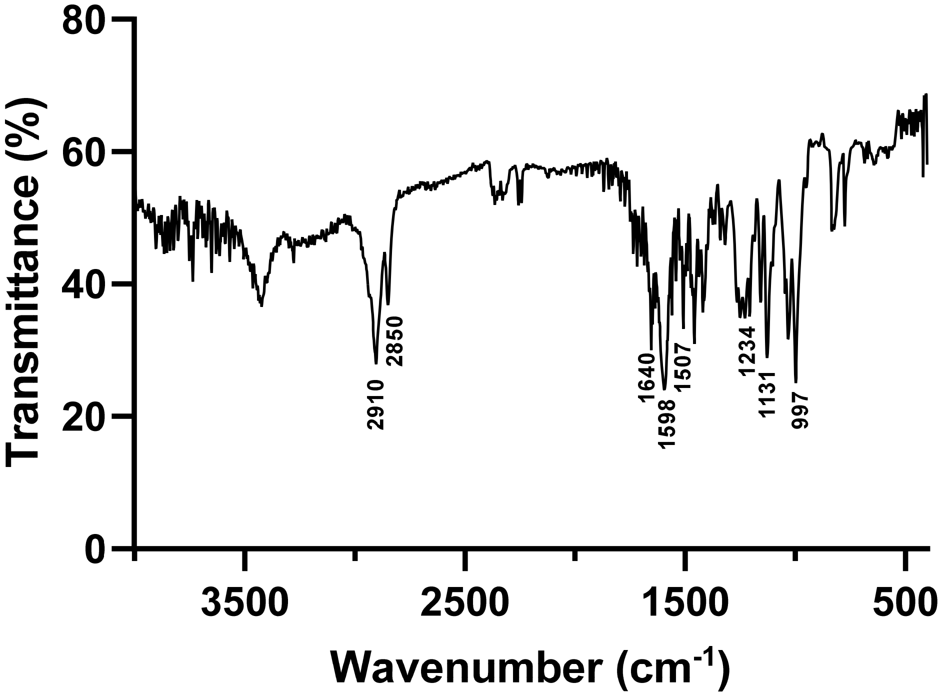


**Figure S24.** IR Spectrum of compound **CA 11**

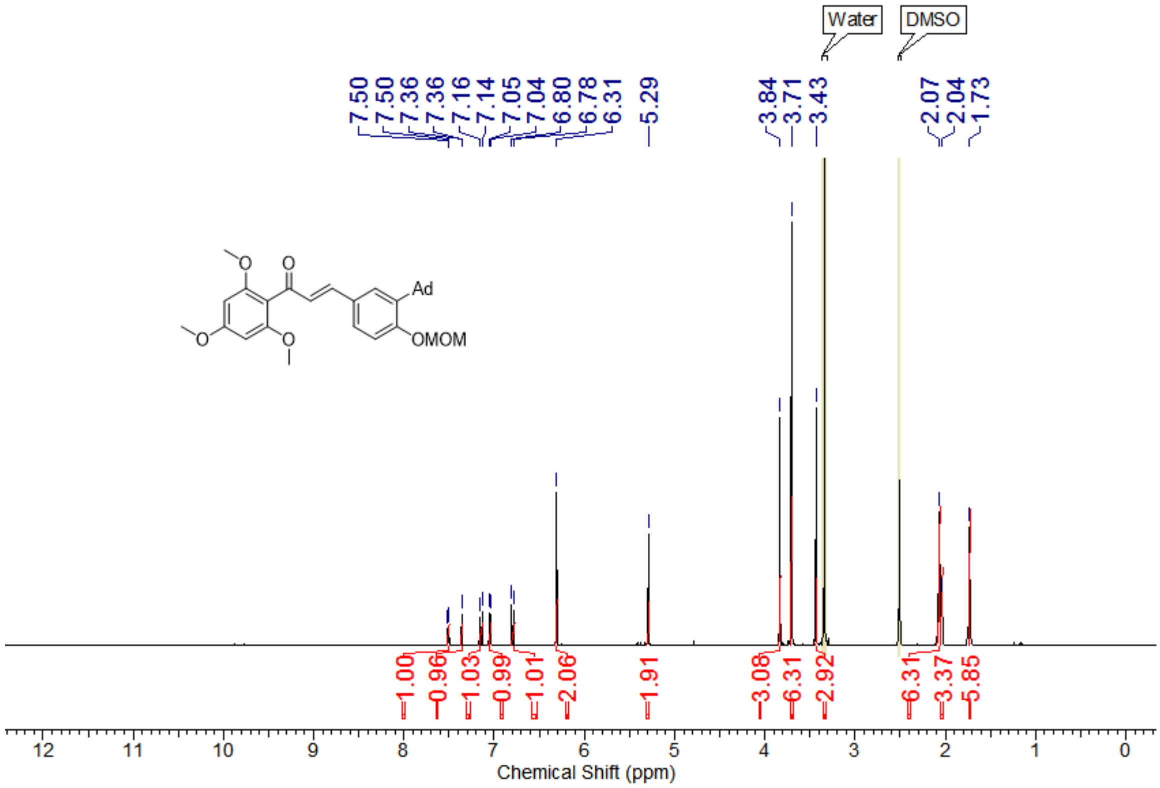


**Figure S25.** $^{1}$H NMR Spectra of compound **CA 12**

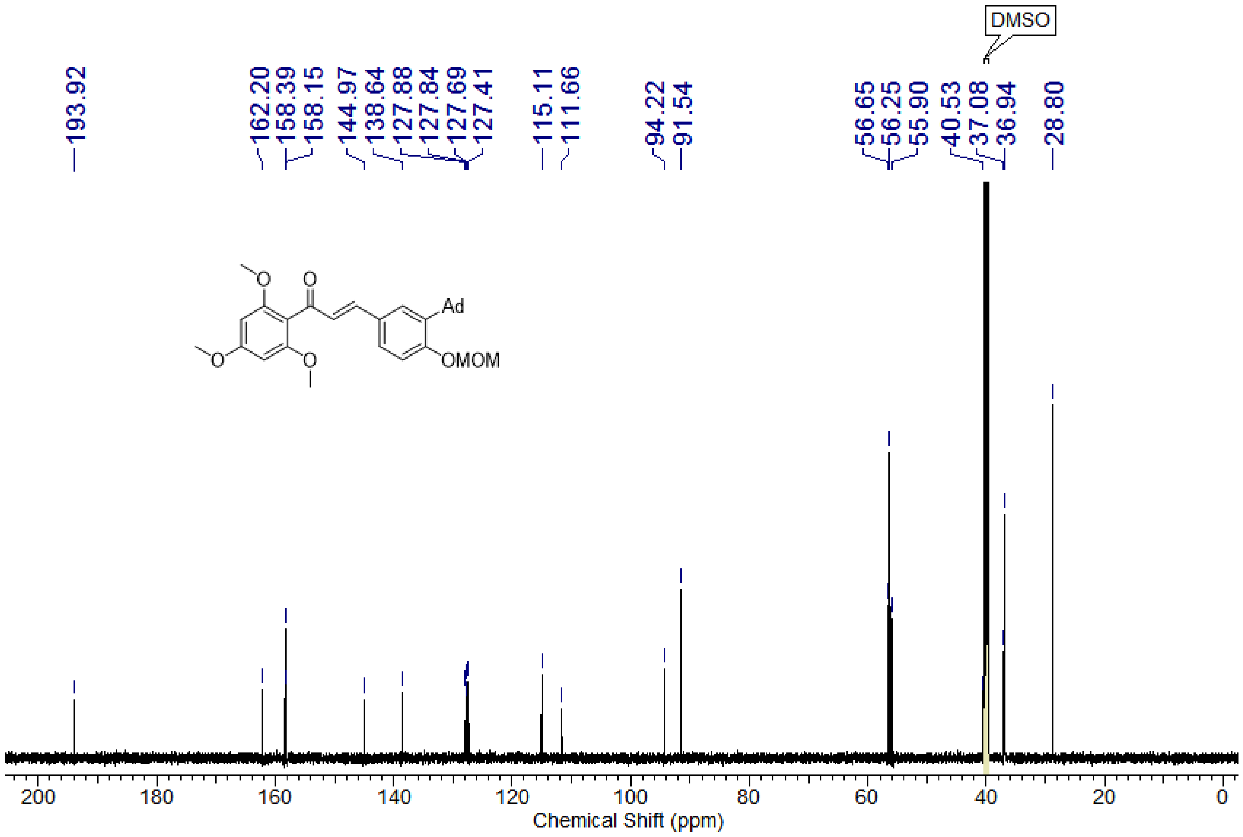


**Figure S26.** $^{13}$C NMR Spectra of compound **CA 12**

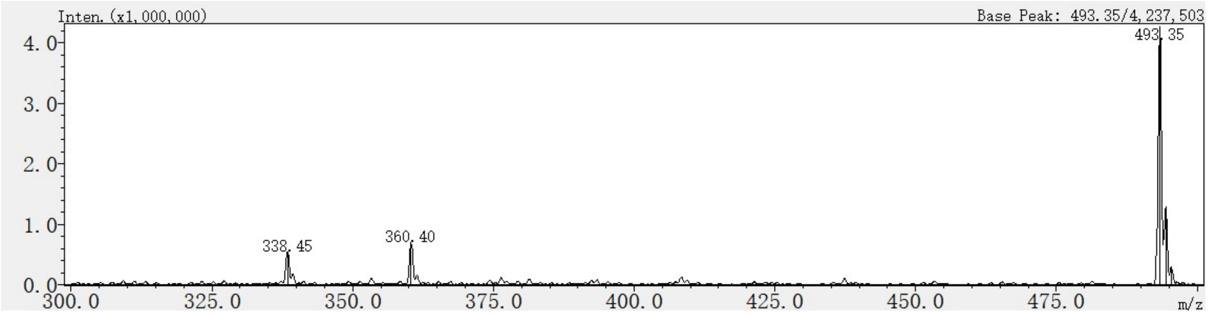


**Figure S27.** Mass Spectrum of compound **CA 12**

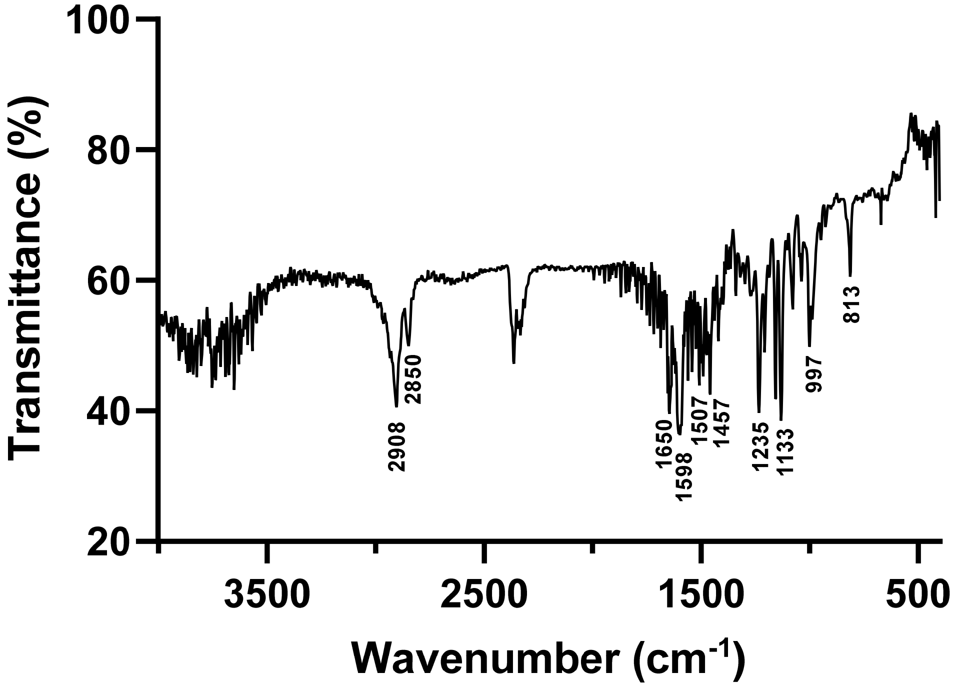


**Figure S28.** IR Spectrum of compound **CA 12**

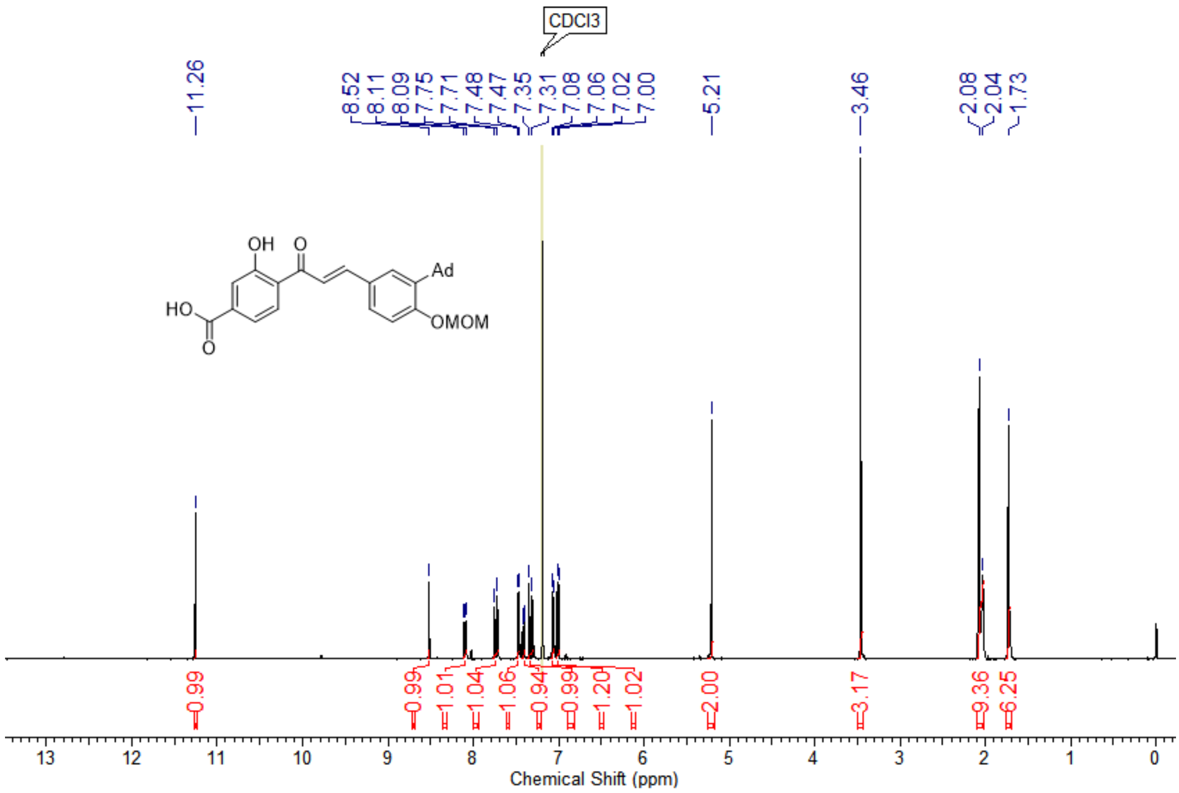


**Figure S29.** $^{1}$H NMR Spectra of compound **CA 13**

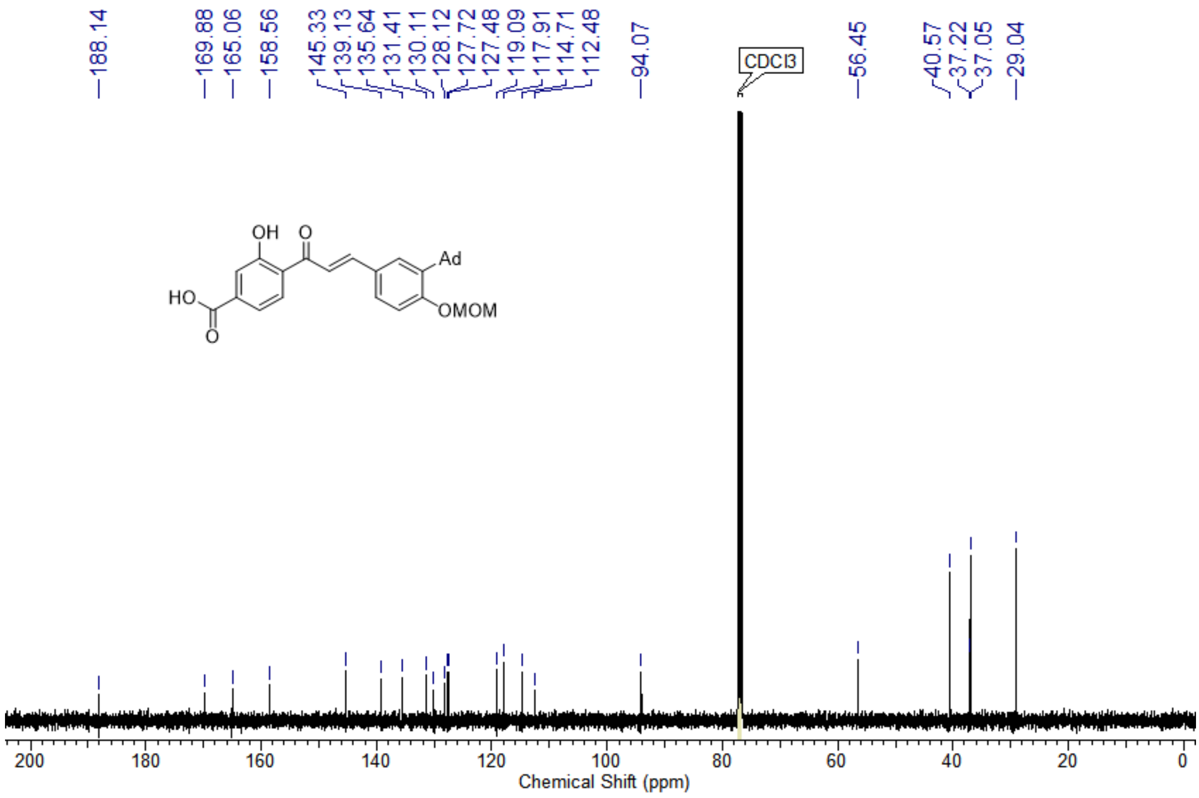


**Figure S30.** $^{13}$C NMR Spectra of compound **CA 13**

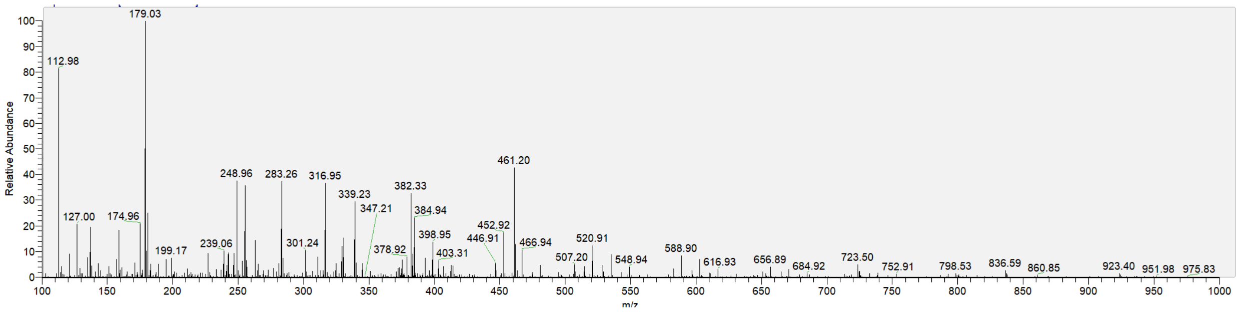


**Figure S31.** Mass Spectrum of compound **CA 13**

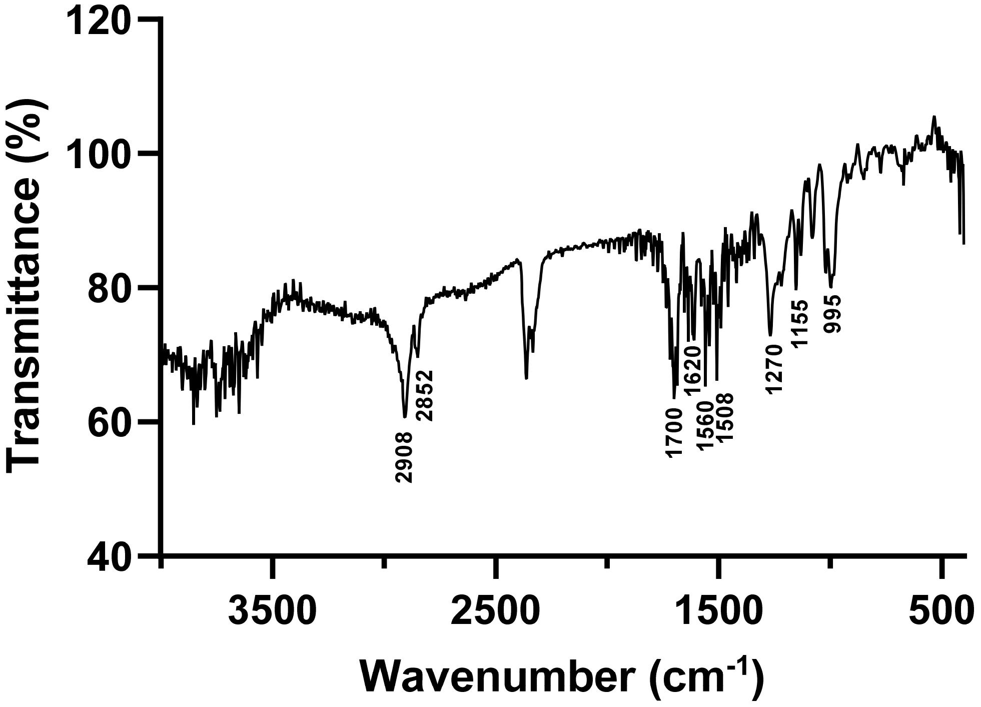


**Figure S32.** IR Spectrum of compound **CA 13**

## Original Western blots

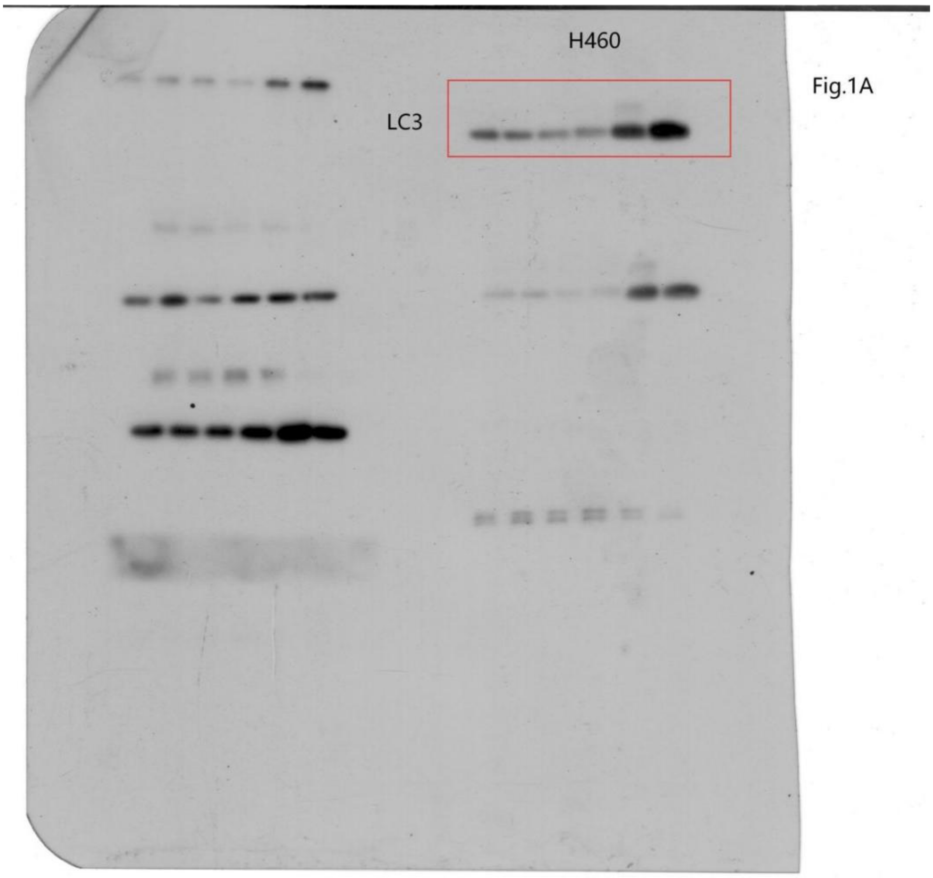


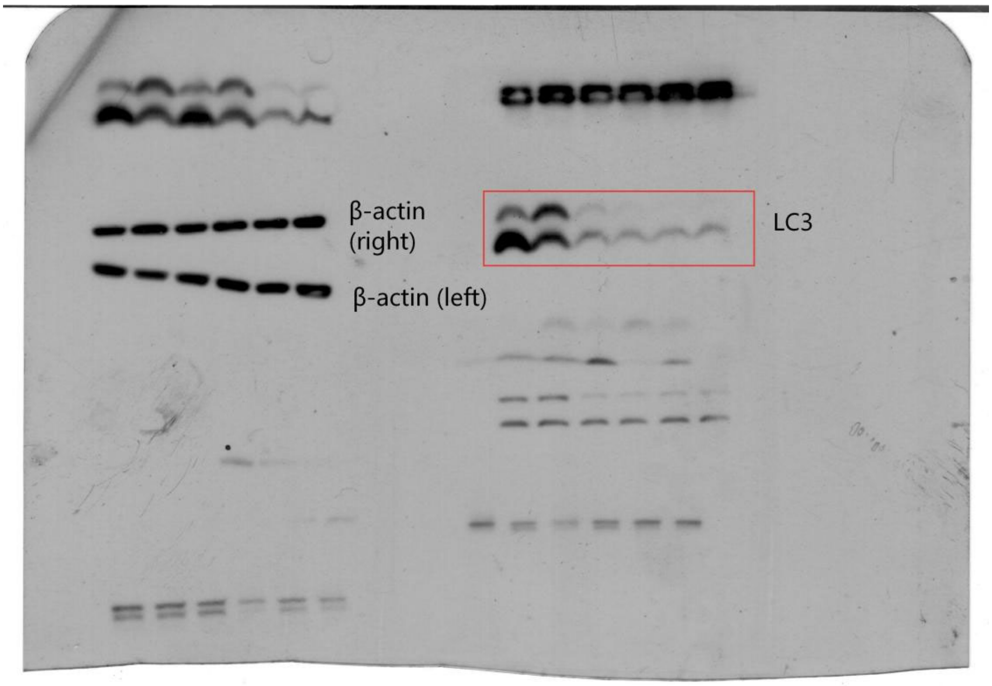


**Western Blot Image of Fig.1A**

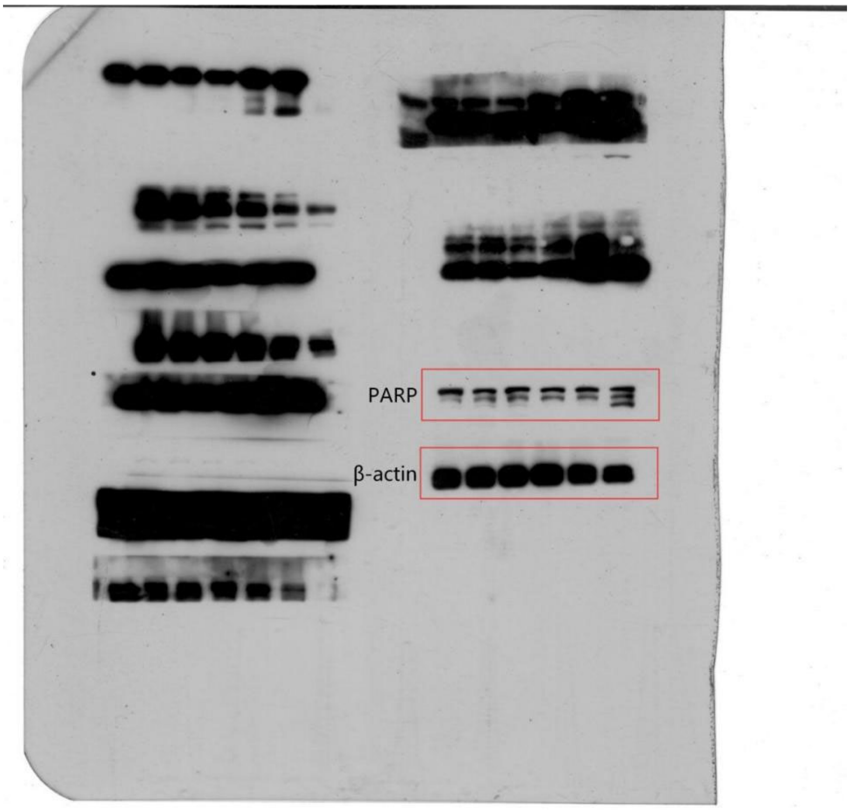


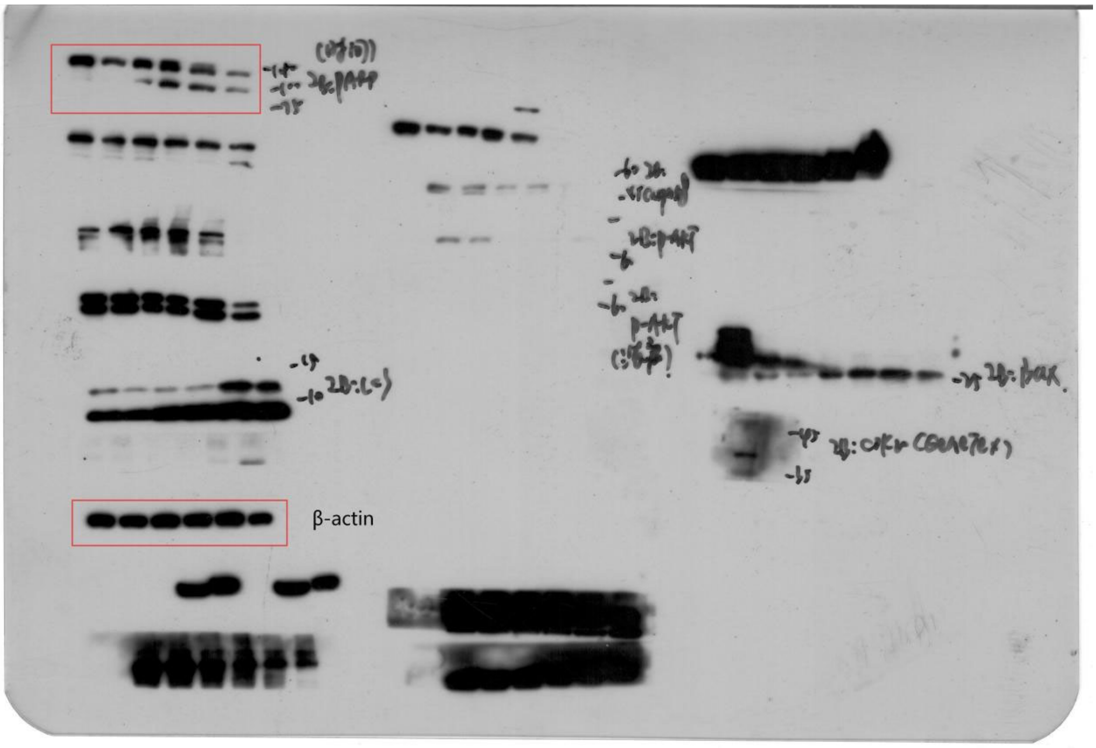


**Western Blot Images of Fig.2A**

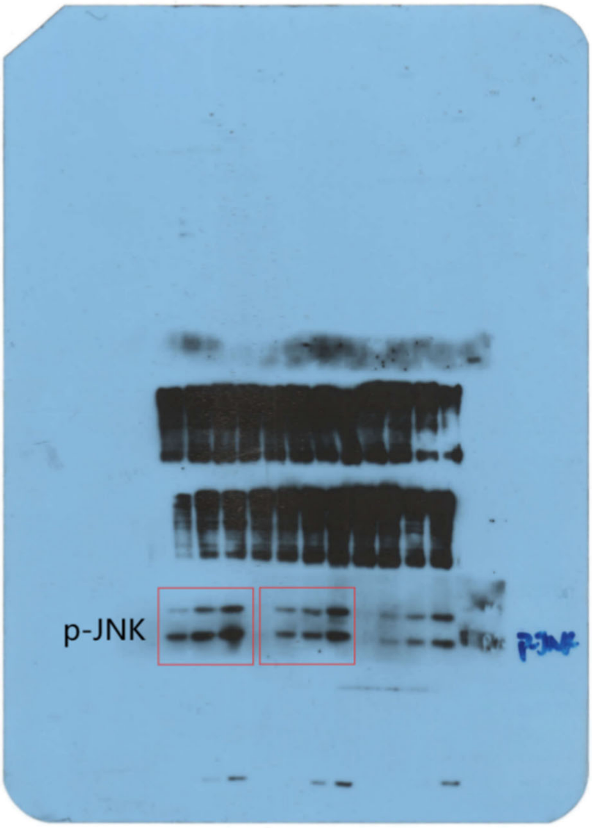


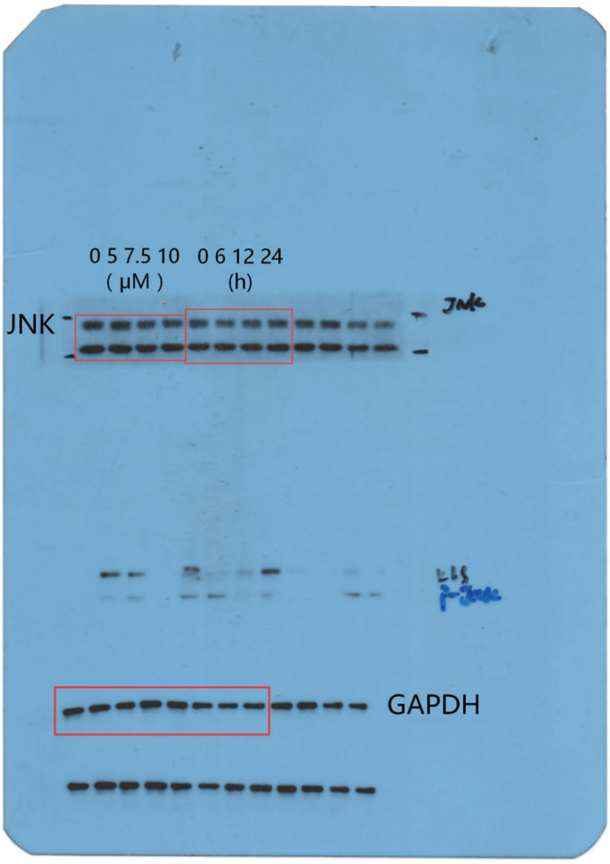


**Western Blot Images of Fig.4A**

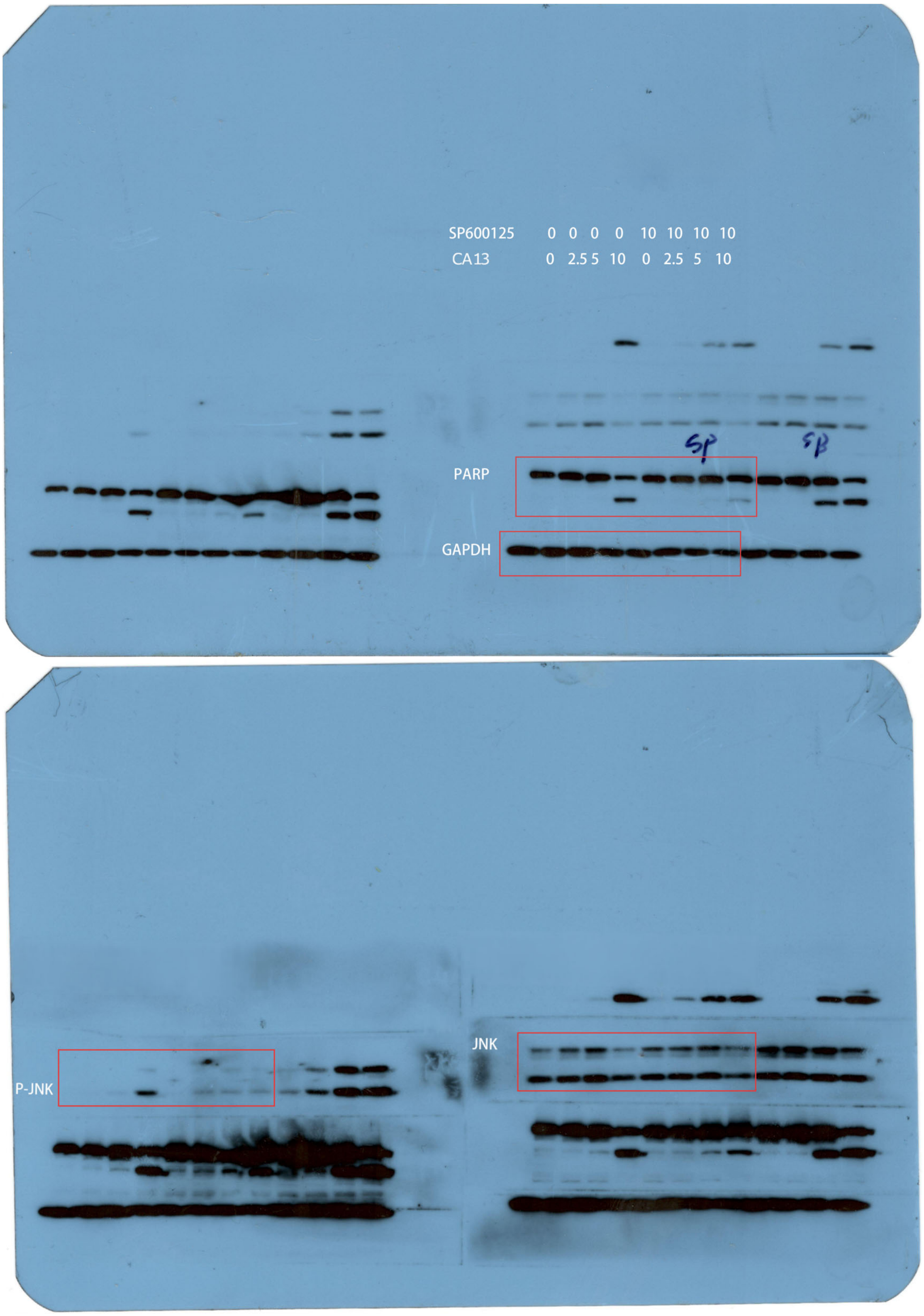


**Western Blot Images of Fig.4C**
